# Psychedelics, Meditation, and Self-Consciousness

**DOI:** 10.3389/fpsyg.2018.01475

**Published:** 2018-09-04

**Authors:** Raphaël Millière, Robin L. Carhart-Harris, Leor Roseman, Fynn-Mathis Trautwein, Aviva Berkovich-Ohana

**Affiliations:** ^1^Faculty of Philosophy, University of Oxford, Oxford, United Kingdom; ^2^Psychedelic Research Group, Psychopharmacology Unit, Department of Medicine, Centre for Psychiatry, Imperial College London, London, United Kingdom; ^3^Department of Social Neuroscience, Max-Planck-Institut für Kognitions- und Neurowissenschaften, Leipzig, Germany; ^4^Faculty of Education, Edmond Safra Brain Research Center, University of Haifa, Haifa, Israel

**Keywords:** psychedelics, meditation, self-consciousness, consciousness, bodily self-consciousness, autobiographical memory, mind wandering, mental time travel

## Abstract

In recent years, the scientific study of meditation and psychedelic drugs has seen remarkable developments. The increased focus on meditation in cognitive neuroscience has led to a cross-cultural classification of standard meditation styles validated by functional and structural neuroanatomical data. Meanwhile, the renaissance of psychedelic research has shed light on the neurophysiology of altered states of consciousness induced by classical psychedelics, such as psilocybin and LSD, whose effects are mainly mediated by agonism of serotonin receptors. Few attempts have been made at bridging these two domains of inquiry, despite intriguing evidence of overlap between the phenomenology and neurophysiology of meditation practice and psychedelic states. In particular, many contemplative traditions explicitly aim at dissolving the sense of self by eliciting altered states of consciousness through meditation, while classical psychedelics are known to produce significant disruptions of self-consciousness, a phenomenon known as drug-induced ego dissolution. In this article, we discuss available evidence regarding convergences and differences between phenomenological and neurophysiological data on meditation practice and psychedelic drug-induced states, with a particular emphasis on alterations of self-experience. While both meditation and psychedelics may disrupt self-consciousness and underlying neural processes, we emphasize that neither meditation nor psychedelic states can be conceived as simple, uniform categories. Moreover, we suggest that there are important phenomenological differences even between conscious states described as experiences of self-loss. As a result, we propose that self-consciousness may be best construed as a multidimensional construct, and that “self-loss,” far from being an unequivocal phenomenon, can take several forms. Indeed, various aspects of self-consciousness, including narrative aspects linked to autobiographical memory, self-related thoughts and mental time travel, and embodied aspects rooted in multisensory processes, may be differently affected by psychedelics and meditation practices. Finally, we consider long-term outcomes of experiences of self-loss induced by meditation and psychedelics on individual traits and prosocial behavior. We call for caution regarding the problematic conflation of temporary states of self-loss with “selflessness” as a behavioral or social trait, although there is preliminary evidence that correlations between short-term experiences of self-loss and long-term trait alterations may exist.

## Introduction

The scientific study of meditation and psychedelic drugs has seen remarkable developments in recent years. The increased focus on meditation in cognitive neuroscience has led to a cross-cultural classification of standard meditation styles validated by functional and structural neuroanatomical data (Lutz et al., 2008; Dahl et al., 2015; Fox et al., 2016). Meanwhile, the renaissance of psychedelic research (Carhart-Harris and Goodwin, 2017) has shed light on the neurophysiology of altered states of consciousness induced by classical hallucinogens, such as psilocybin and LSD, whose effects are mainly mediated by agonism of serotonin receptors, and the serotonin 2A receptor subtype specifically (Carhart-Harris et al., 2012, 2016b; Nichols, 2016). However, few attempts have been made at bridging these two domains of inquiry, despite increasing evidence of overlap between the phenomenology and neurophysiology of meditation practices and psychedelic states.

In particular, many contemplative traditions explicitly aim at dissolving the sense of self by eliciting altered states of consciousness through meditation (Austin, 2000; Josipovic, 2010; Vago and Silbersweig, 2012; Dahl et al., 2015), while classical psychedelics are known to produce significant disruptions of self-consciousness^1^, a phenomenon known as “drug-induced ego dissolution” (Millière, 2017; Nour and Carhart-Harris, 2017; DIED). In this article, we discuss available evidence regarding convergences and differences between phenomenological and neurophysiological data on meditation practice and psychedelic drug-induced states, with a particular emphasis on alterations of self-experience. While both meditation and psychedelics are suspected to disrupt self-consciousness and its underlying neural processes, this general hypothesis requires careful qualification.

First, it is important to emphasize right away that neither meditation nor psychedelic states can be conceived as simple, uniform categories. Many variables modulate the subjective effects of contemplative practice and psychedelics, including the style of meditation or the drug and dosage used, as well as personal factors such as level of experience and personality traits. In particular, dramatic disruptions of self-consciousness seem to occur mostly for highly experienced meditators or with high doses of psychedelics. Thus, we suggest that both meditation and psychedelics can induce a wide variety of global states of consciousness, but these states are sensitive to a multitude of factors in addition to the specific inducers we are highlighting here^2^.

In addition, we suggest that there are important phenomenological differences even between conscious states described as experiences of self-loss. As a result, we propose that self-consciousness may be best construed as a multidimensional construct, and that “self-loss” or “ego dissolution,” far from being an unequivocal phenomenon, can take several forms. Indeed, various aspects of self-consciousness, including narrative aspects linked to autobiographical memory, self-related thoughts and mental time travel, and embodied aspects rooted in multisensory processes, may be differently affected by psychedelics and meditation practices. It is also worth acknowledging that “self-loss” or “ego dissolution” may be a non-linear phenomenon that only occurs after a critical inflection point has been reached.

Finally, we consider long-term outcomes of experiences of self-loss induced by meditation and psychedelics on individual traits and prosocial behavior. We call for caution regarding the problematic conflation of temporary states of self-loss with “selflessness” as a personal or social trait, although it remains possible that correlations exist between short-term experiences and long-term dispositions in this regard.

## The neuroscience of meditation and psychedelics: an overview

Meditation refers to a set of cognitive training techniques and practices that aim to monitor and regulate attention, perception, emotion and homeostasis (e.g., breathing rate) (Fox and Cahn, forthcoming; Tang et al., 2015). Such techniques and practices have been developed in many different cultures and spiritual traditions, yielding more than a 100 varieties of meditation. Most scientific research on the topic has focused on techniques originating in the Buddhist tradition from China, Tibet, India and Southeast Asia, with a particular focus on practices often subsumed under the loose category of mindfulness meditation (see Box 1; on the limitations of the notion of mindfulness, see Dam et al., 2018). Nonetheless, there are a growing number of studies on meditative practices from other contemplative traditions – including yogic, Hindu, Christian, Sufi, shamanic and transcendental practices.

Box 1 **Glossary.**

**Drug-induced Ego Dissolution (DIED)**. A family of acute effects produced by high doses of psychedelic drugs, typically reported as a loss of one's sense of self and self-world boundary.

**Focused Attention (FA)**. A common style of meditation that involves sustaining one's attentional focus on a particular object, either internal (e.g., breathing) or external (e.g., a candle flame). The practitioner is instructed to monitor their attention, notice episodes of distraction (mind-wandering), and bring their attention back to the object. FA is usually the starting point for novice meditators.

**Loving-Kindness Meditation (LK)**. A common style of meditation that focuses on developing compassion and love for oneself and others, gradually extending the focus of empathy to foreign and disliked individuals or even all living-beings. While loving-kindness meditation incorporates technical elements from FA and open monitoring (OM, defined below), it has a distinct phenomenology and neural correlates due to its emotional content.

**Mantra Recitation (MR)**. A style of meditation that involves repeating a sound, word or sentence, either aloud or in one's mind, in order to calm the mind and avoid mind-wandering. Although MR is arguably a form of Focused Attention meditation, it is distinguished by its speech component and may have distinct neural correlates.

**Meta-Awareness**. The ability to take note of the content of one's current mental state. In the context of meditative practices, meta-awareness often refers specifically to the meditator's awareness of episodes of mind-wandering (spontaneous thoughts arising during meditation).

**Mindfulness Meditation**. A group of practices aimed at cultivating mindfulness, typically defined as a state of non-judgmental awareness to one's present moment experience. Mindfulness meditation may refer to both focused attention (FA) and open monitoring (OM) practices.

**Non-Dual Awareness (NDA)**. In many contemplative traditions (including Advaita Vedanta and Kashmiri Shaivism within Hinduism, and Dzogchen and Mahāmudrā within Buddhism), the practice of meditation aims at recognizing the illusory nature of the subject-object dichotomy that allegedly structures ordinary conscious experience, thus revealing the “non-dual awareness” that lies at the background of consciousness.

**Open Monitoring (OM)**. A common style of meditation that aims at bringing attention to the present moment and openly observing mental contents without getting caught up in focusing on any of them. Open monitoring meditation traditionally follows focused awareness, as the practitioner learns to switch from a narrow attentional focus on an object to a global awareness of the present moment.

**Psychedelic Drugs**. A family of psychoactive compounds whose complex effects on the quality of conscious experience are mainly mediated by their action on serotonin receptors in the brain (and specifically their binding to and stimulation of serotonin 2A receptor subtype). Psychedelic substances include mescaline, psilocybin (so-called “magic mushrooms”), Lysergic Acid Diethylamide (LSD), N,N-Dimethyltryptamine (DMT), and the DMT-containing brew Ayahuasca.

**Pure Consciousness (PC)**. A state of consciousness described as “objectless” or entirely devoid of phenomenal content. While the possibility of such states is very controversial, certain conscious states induced by some meditative practices and classical psychedelics might lack at least *ordinary* phenomenal content. In the Hindu and Buddhist traditions, the practice of *Samadhi* is often described as leading to the experience of PC.

To address this remarkable diversity of traditions, researchers have also sought to categorize the main styles of meditation across cultural, geographical and historical contexts, based on the core goals and principles of the mental techniques involved (Lutz et al., 2008; Travis and Shear, 2010; Nash and Newberg, 2013). Common styles of meditation include focused attention meditation (FA)—which requires sustaining one's attention on a particular object or sensation such as the breath—, open monitoring meditation (OM)—which involves a non-judgmental, non-selective awareness of the present moment—, loving-kindness and compassion meditation (LK)—which involves the cultivation of compassion toward oneself and others—, and mantra recitation (MR)—which involves the repetition of a sound, word or sentence (see Box 1). The subjective effects of meditation are multifaceted, including enhanced attention and sensory processing (Brown, 1977; Jha et al., 2007), largely positive emotions and mood (Ortner et al., 2007; Davidson and Lutz, 2008; Lutz et al., 2008), increased cognitive flexibility and creativity (Horan, 2009; Capurso et al., 2014; Ding et al., 2014; Berkovich-Ohana et al., 2017), and, in some cases (usually with increasing expertise) dramatic disruptions of one's sense of self (Austin, 2006; Dorjee, 2016; Berkovich-Ohana and Wittmann, 2017).

Classic psychedelics, also known as classic hallucinogens, are a family of psychoactive substances that exert their subjective effects primarily by agonism (or partial agonism) of serotonin 2A (5-HT2A) receptors (Halberstadt, 2015; Nichols, 2016). Psychedelics include molecules found in the natural world such as psilocybin (a prodrug of psilocin found in so-called “magic mushrooms”), mescaline (found in various South American cacti, including Peyote, San Pedro and Peruvian torch), N,N-Dimethyltryptamine (DMT, found in many plants such as *Mimosa tenuiflora, Diplopterys cabrerana*, and *Psychotria viridis*, and used in combination with monoamine oxidase inhibitors in the shamanic brew Ayahuasca) and 5-MeO-DMT (found in the toxin of the toad *Bufo alvarius*, as well as a wide variety of plants such as *Anadenanthera peregrina* used in yopo snuff). Psychedelics also include many synthetic compounds, such as lysergic acid diethylamide (LSD) and phenethylamines of the 2C-*x* family (e.g., 2C-B). The subjective effects of psychedelics are complex and multifaceted, including visual and auditory distortions and complex closed-eye “visions,” profound changes in emotions and mood, heightened sensitivity to internal and external context, and at higher doses, dramatic alterations of self-consciousness known as drug-induced ego dissolution (DIED) (Strassman et al., 1994; Vollenweider et al., 1998; Studerus et al., 2011; Carhart-Harris et al., 2012, 2016b; Schmid et al., 2015; Fox et al., 2016; Millière, 2017).

### Neural correlates of meditative practices

Over the past 20 years, a large number of neuroimaging studies have investigated various styles of meditation, using electro-encephalography (EEG), functional magnetic resonance imaging (fMRI) and positron emission tomography (PET) to measure changes in blood flow and electrical activity in the brain during meditation. This research program seeking to isolate the neural correlates of various meditation practices has come to be known as contemplative neuroscience. Interestingly, the wealth of data collected from these studies has begun to reveal that meditation practices from distinct traditions relying on similar mental techniques, also share some common neural correlates. A recent meta-analysis of 78 functional neuroimaging studies of meditation found dissociable patterns of activation and deactivation for four common styles of meditation: focused attention (FA), mantra recitation (MR), open monitoring (OM), and loving-kindness meditation (LK) (Fox et al., 2016; see Table 1).

**Table 1:** Significant clusters of activation/deactivation in four common meditation styles from a meta-analysis of 78 neuroimaging studies (Fox et al., 2016).

	**Focused Attention (FA)**	**Mantra Recitation (MR)**	**Open Monitoring (OM)**	**Loving-Kindness (LK)**
Significant activation clusters	PMC Dorsal ACC	Broca's Area PMC SMC	Insula IFG SMA/pre-SMA PMC	IPL Anterior Insula
Significant deactivation clusters	PCC IPL	Anterior Insula	Thalamus	/

FA was correlated with significant activation clusters in executive brain areas such as the premotor cortex and the dorsal anterior cingulate cortex, which may underlie top-down regulation of attention and monitoring of spontaneous thoughts. FA was also correlated with the deactivation of two important hubs of the so-called default-mode network (DMN) namely, the posterior cingulate cortex and inferior parietal lobule, involved in a plethora of introspection-related functions including self-reflection, mind-wandering, autobiographical memory recollection, mental time travel to the future and imagination more broadly. The main clusters of activation for MR were found in the motor control network (Broca's area, premotor cortex and supplementary motor cortex) and the putamen. The involvement of these regions in speech production and attention regulation is consistent with the practice of mantra recitation, which relies on the constant repetition of a phrase either in one's mind or out loud. MR was also associated with a significant deactivation cluster in the anterior insula, suggesting that the focus on a repeated phrase is linked to decreased awareness of bodily sensation.

By contrast, OM was found to be correlated with activation clusters in the insular cortex, involved in awareness of interoceptive signals, as well as in brain regions associated with the voluntary control of thought and action, such as the left inferior frontal gyrus, the pre-supplementary and supplementary motor areas, and the premotor cortex. OM was also associated with the deactivation of the right thalamus, a region involved in filtering out sensory stimuli (sensory gating), suggesting that the increased focus of awareness during OM is mediated by decreased sensory gating. Finally, significant activation clusters for LK were found in the right somatosensory cortices, the inferior parietal lobule and the right anterior insula, while no significant deactivation cluster was found. Although these four meditation styles are clearly dissociated by their neural correlates, Fox and colleagues found a few recurrent patterns of activity modulation, in particular in the insular cortex, an important multisensory area heavily involved in interoceptive awareness (Craig, 2002; Simmons et al., 2013).

It is worth noting that distinct styles of meditation were found to modulate the activity of the insula in different ways, namely activation for FA, OM, and LK, and deactivation for MR. Nonetheless, the involvement of the insula in all four styles of meditation points toward the central role of the attentional control of bodily awareness, and awareness of breathing in particular, during various contemplative practices. Other convergent patterns of activity were found to a lesser extent in regions involved in the regulation of attention such as the premotor cortex, supplementary motor area and dorsal cingulate cortex (Fox et al., 2016).

Importantly, and albeit not emphasized by Fox et al. (2016), attenuation of either activity or functional connectivity in the default mode network (DMN), a large-scale intrinsic network which is highly active at rest but less active during goal-directed tasks (Raichle et al., 2001), was shown for MR (Berkovich-Ohana et al., 2015), and in many studies of mindfulness meditation (combining FA and OM in different degrees). These studies reported a decrease of activity in key nodes of the DMN, particularly in the medial prefrontal cortex (Farb et al., 2007, 2013; Brewer et al., 2011; Scheibner et al., 2017) and in the posterior cingulate cortex (Brewer et al., 2011; Ives-Deliperi et al., 2011; Pagnoni, 2012; Brewer and Garrison, 2014; Lutz et al., 2016; Scheibner et al., 2017), compared to meditation-naïve controls or within-group resting state (reviewed by Tang et al., 2015). While DMN deactivation is not specific to mindfulness meditation, it has been found to be more pronounced during meditation than during other cognitive tasks (Garrison et al., 2015). Moreover, several studies reported altered connectivity during different types of meditation most consistently in association with the DMN (Tang et al., 2015). Specifically, within-DMN connectivity was found to be reduced during LK (Garrison et al., 2014).

### Neural correlates of psychedelic states

Recent years have seen a renaissance of scientific research on psychedelic drugs, using modern neuroimaging techniques to gather insight into the neural correlates of their vivid subjective effects (see dos Santos et al., 2016 for a review). Early neuroimaging studies using PET and single-photon emission computed tomography (SPECT) after administration of psilocybin and mescaline found excitatory effects on frontal cortical areas, medial temporal lobes and the amygdala (Hermle et al., 1992; Vollenweider et al., 1997, 1998; Gouzoulis-Mayfrank et al., 1999). By contrast, recent fMRI of psilocybin and ayahuasca found significant *reductions* in activity across many brain areas, including frontal and temporal cortical regions, as well as hubs of the DMN (Carhart-Harris et al., 2012; Palhano-Fontes et al., 2015; see Table 2). This apparent discrepancy could be due to the much greater timescales used by PET/SPECT compared to fMRI (Carhart-Harris et al., 2012).

**Table 2:** Main changes in activity and connectivity in the psychedelic state.

**References**	**Psychedelic drug**	**Changes in activity**	**Changes in functional connectivity**
Carhart-Harris et al., 2012	Psilocybin	↘ in ACC, PCC, mPFC and thalamus	↘ mPFC-PCC connectivity
de Araujo et al., 2012	Ayahuasca	↗ in primary visual cortex during imagery task ↗ cuneus, lingual gyrus, PH, RSC, and frontopolar cortex	↗ DMN-TPN connectivity
Carhart-Harris et al., 2013	Psilocybin		↗ DMN-TPN connectivity
Roseman et al., 2014	Psilocybin		↗ between-network connectivity
Tagliazucchi et al., 2014	Psilocybin		↗ diversity of connectivity motifs in HP-ACC network
Palhano-Fontes et al., 2015	Ayahuasca	↘ in mPFC, PCC, precuneus	↘ PCC-precuneus connectivity
Carhart-Harris et al., 2016b	LSD	↗ in visual cortex (correlating with visual hallucinations)	↘ DMN integrity and PHC-RSC connectivity
Tagliazucchi et al., 2016	LSD		↗ global connectivity, especially thalamus, frontoparietal and inferior temporal cortices
Müller et al., 2017	LSD		↗ thalamocortical connectivity

Recent fMRI studies also revealed alterations of resting-state functional connectivity in key nodes of the DMN after administration of psilocybin (Carhart-Harris et al., 2012, 2013; Tagliazucchi et al., 2014; Lebedev et al., 2015), ayahuasca (Palhano-Fontes et al., 2015) and LSD (Carhart-Harris et al., 2016b; Tagliazucchi et al., 2016; see Table 2). Researchers found increased integration between cortical regions under ayahuasca (de Araujo et al., 2012), psilocybin (Carhart-Harris et al., 2013; Roseman et al., 2014), and LSD (Carhart-Harris et al., 2016b; Tagliazucchi et al., 2016; Müller et al., 2017). Finally, psilocybin and LSD were found to produce an enhanced repertoire of dynamical brain states (Tagliazucchi et al., 2014; Atasoy et al., 2017) and increased spontaneous MEG signal diversity (Schartner et al., 2017).

Psychedelic drugs are known to produce short-term, dramatic effects on self-consciousness, especially at higher doses. This phenomenon is known as drug-induced ego dissolution (DIED); it is described a loss of one's sense of self and self-world boundaries, together with a concomitant oceanic feeling of “oneness” or “unity” (Letheby and Gerrans, 2017; Millière, 2017; Nour and Carhart-Harris, 2017). Such effects on self-consciousness had already been reported in early studies with mescaline (Mayer-Gross and Stein, 1926; Beringer, 1927; Guttmann and Maclay, 1937), LSD (Anderson and Rawnsley, 1954; Savage, 1955; Von Mering et al., 1955; Bercel et al., 1956; Klee, 1963; Sedman and Kenna, 1964; Pahnke and Richards, 1966) and psilocybin (Rümmele and Gnirss, 1961; Pahnke, 1966). Recent studies have sought to investigate the neural correlates of DIED, using correlations between questionnaire items and neuroimaging data. The main correlates of DIED, summarized in Table 3, include increased functional connectivity between the DMN and a task-positive network (Carhart-Harris et al., 2013), decreased integrity of the DMN (i.e., decreased within network correlation of timecourses) (Carhart-Harris et al., 2016b) and salience network (Lebedev et al., 2015), decreased connectivity between the parahippocampal and retrosplenial cortices (Carhart-Harris et al., 2016b), increased entropic brain activity (Lebedev et al., 2016) and spontaneous MEG signal diversity (Schartner et al., 2017), and decreased mean energy and fluctuations of low frequency connectome harmonics (Atasoy et al., 2017).

**Table 3:** Neural correlates of drug-induced ego dissolution (DIED).

**References**	**Drug**	**Correlates of DIED**
Carhart-Harris et al., 2013	Psilocybin	↗ Increased DMN-TPN functional connectivity
Muthukumaraswamy et al., 2013	Psilocybin	↘ alpha power in PCC
Lebedev et al., 2015	Psilocybin	↘ functional connectivity in aPHC ↘ MTL-neocortex coupling ↘ integrity of salience network ↘ interhemispheric connectivity
Tagliazucchi et al., 2016	LSD	↗ Connectivity density in bilateral TPJ/angular gyrus and insular cortex
Carhart-Harris et al., 2016b	LSD	↘ integrity of DMN ↘ PHC-RSC connectivity ↘ alpha & delta power in PCC
Lebedev et al., 2016	LSD	↗ entropic brain activity
Schartner et al., 2017	Psilocybin	↗ spontaneous MEG signal diversity (measured with Lempel-Ziv complexity)
Atasoy et al., 2017	LSD	↘ mean energy and energy fluctuations of low frequency connectome harmonics

## Alterations of self-consciousness induced by meditation and psychedelics

While it is often claimed that meditation and psychedelic drugs are both able to induce “selfless” states of consciousness, that is conscious states entirely lacking a sense of self, this statement requires qualification. Indeed, “self-loss” and related expressions such as “ego dissolution” are notoriously ambiguous notions. Self-consciousness itself may be best construed as a multidimensional construct including somatosensory, agentive, narrative and social components (Gallagher, 2000, 2013; Zahavi, 2014; Seth and Friston, 2016). A conscious state in which one of these aspects is radically disrupted may be described as “selfless” in one respect, although a subject undergoing such a state could retain other forms of self-consciousness. Therefore, it is important to investigate which aspect(s) of self-consciousness can be disrupted by meditation and psychedelics, and whether both modes of induction may alter self-consciousness in similar ways. In addition, there is a further question regarding the possibility of truly “selfless” states, namely conscious mental states lacking *any* kind of self-consciousness. While some meditative practices and psychedelic drugs have been hypothesized to produce such “selfless” states, this claim needs to be supported by an examination of phenomenological reports in light of the distinction between different aspects of self-consciousness. In this section, we discuss the ways in which meditation and psychedelics may disrupt narrative, agentive and somatosensory aspects of self-consciousness, either in isolation or in combination. Moreover, we critically examine theoretical discussions and self-reports about states of “non-dual awareness” and “pure consciousness” allegedly induced by meditation.

### Disruption of narrative aspects of self-consciousness

The familiar experience of thinking about oneself is perhaps the most salient form of self-consciousness. In philosophy of mind, this is also known as having *de se* thoughts, namely thoughts that involve the first-person concept and are naturally expressed using the first-person pronoun (García-Carpintero, 2015). *De se* thoughts themselves come in different flavors, which are more or less egocentric. Thus, one may explicitly reflect on one's personality traits or one's life trajectory, both of which are important elements of an individual's identity. This category of *de se* thought broadly pertains to the entertainment of core self-related beliefs, and is often linked to the notion of narrative selfhood—the stories we tell ourselves about the kind of person we are or want to be. Admittedly, this kind of *de se* thought only occurs sporadically in the waking state, because we are not constantly reflecting on our personal identity. However, *de se* thoughts also include more mundane and pervasive instances of mind-wandering, such as wondering what one will have for dinner. Such thoughts also link back to the self, insofar as they engage the first-person concept, even if they are not directly related to fundamental beliefs about one's identity. More generally, this family of self-referential cognitive content includes not only *de se* thoughts about the present moment, but also autobiographical memory retrieval and self-centered mental time travel to the future, both of which also crucially involve the self. Together, these self-referential mental episodes constitute what may be called narrative self-consciousness, namely the complex sequences of self-centered thoughts, memories and imaginings that weave the narrative of our daily lives and shape our core self-related beliefs (Damasio, 1999; Gallagher, 2000; Schechtman, 2011).

There are at least two ways in which narrative self-consciousness may be disrupted. First, the rate of occurrence of self-referential thought and mental time travel may be dramatically reduced, or altogether suppressed, during a certain time interval. There is convincing evidence from experience sampling studies that mind-wandering and mental time travel is more ubiquitous in the waking state than we might think, due to the fact that we are often unaware of such episodes (Smallwood and Schooler, 2006; Killingsworth and Gilbert, 2010; Baird et al., 2011). Moreover, daily mind-wandering episodes are predominantly future-focused, and frequently involve the planning and anticipation of personal goals, known as autobiographical planning (Baird et al., 2011; D'Argembeau, 2016). Thus, it is not exaggerated to say that a large part of our lives is spent entertaining self-involving thoughts, insofar as this includes spontaneous *de se* thoughts about the future. Most contemplative practices, especially in the Buddhist tradition, explicitly aim at increasing meta-awareness of mind-wandering, namely monitoring and taking explicit note of spontaneous thoughts, in order to disengage from them and re-focus attention back onto a particular object (such as the breath or a mantra) or on a wider awareness of the present moment (Hasenkamp, 2018). Once attention has been stabilized and the mind “quieted,” meditators can undergo prolonged conscious episodes entirely lacking in self-referential thoughts^3^.

This is consistent with the neurophysiological basis of mindfulness meditation reviewed in the previous section. While key nodes of the DMN such as the mPFC and the PCC are especially active during mind-wandering (Christoff et al., 2009; Fox et al., 2015), a number of studies have found that these regions are deactivated during mindfulness meditation—which is consistent with the practice's focus on attentional control of mind-wandering episodes (Farb et al., 2007; Brewer et al., 2011; Lutz et al., 2016; Scheibner et al., 2017). Importantly, the decrease in DMN activity is more significant during meditation than during other cognitive tasks (Garrison et al., 2015), which speaks to the crucial issue of specificity and may be characteristic of the suppression of mind-wandering that can be achieved by trained meditators. It is also worth noting that experiences of flow (Csikszentmihalyi, 1975, 1990), namely states of intense focus in which self-referential processing is inhibited (such as intense practice in expert athletes or jazz improvisation in expert musicians), are also associated with decreased activity in the mPFC (Ulrich et al., 2014). Interestingly, psychedelic drugs have been shown to decrease activity in the mPFC and PCC as well (Carhart-Harris et al., 2012; Palhano-Fontes et al., 2015). These nodes of the DMN have been shown to be more active during various types of self-related stimuli (Northoff et al., 2006; Sugiura et al., 2008; Qin and Northoff, 2011; Tacikowski et al., 2017), self-reflection (Jenkins and Mitchell, 2011; D'Argembeau, 2018) and autobiographical memory retrieval (Cabeza and St Jacques, 2007). A recent study using dynamic causal modeling suggested that self-referential processes are driven by PCC activity and modulated by the regulatory influences of the mPFC (Davey et al., 2016).

Aside from changes in cerebral blood flow in these regions, psilocybin, ayahuasca and LSD have also been consistently linked to decreased functional integrity of the DMN (Carhart-Harris et al., 2012, 2016b; Palhano-Fontes et al., 2015). Moreover, DMN disintegration was correlated with reports of ego dissolution (Carhart-Harris et al., 2016b) and decreased mental time travel to the past (Speth et al., 2016). These findings are intriguing, because strong DMN connectivity at rest is associated with increased tendency for mental time travel, in particular spontaneous thoughts about the future (Schacter et al., 2007; Godwin et al., 2017; Karapanagiotidis et al., 2017; Wang et al., 2017). This suggests that reports of drug-induced ego dissolution may be related to the experience of decreased self-referential thought and mental time travel, which is also fundamental to the practice of meditation.

While the temporary cessation of self-referential thoughts is one way in which narrative self-consciousness may be altered, it may also be disrupted by a total loss of access to autobiographical memories and self-related beliefs. These two cases are not unrelated, given that the unavailability of personal memories and beliefs precludes the possibility of at least some forms of *de se* thought and mental time travel. However, the experience of losing access to these memories and beliefs might differ from the mere cessation of *de se* thought from a phenomenological point of view. Indeed, anecdotal evidence from narrative self-reports suggests that drug-induced ego dissolution may be related in some cases to reversible retrograde amnesia, specifically regarding abstract information about oneself (i.e., semantic autobiographical memory). For example, one subject responding to an online questionnaire on ego dissolution reported “forgetting that I was a male, a human, a being on Earth—all gone, just infinite sensations and visions,” while another stated “I no longer felt human. I didn't remember what a human was” (R.M., unpublished data from an online survey on drug-induced ego dissolution completed by experienced users of psychedelic drugs)^4^.

Interestingly, decreased functional connectivity within the DMN is associated with age-related memory deficits (Damoiseaux et al., 2008; Ward et al., 2015) and Alzheimer's disease (Greicius et al., 2004; Dennis and Thompson, 2014; Dillen et al., 2017). Given the link between DMN disintegration (decreased within-network connectivity) and ego dissolution, it is intriguing to speculate that the temporary loss of access to semantic autobiographical information that can occur in the psychedelic state may be mediated by pronounced reductions in DMN integrity. Interestingly, *post-hoc* mediation analyses from Dillen and colleagues revealed that the retrosplenial cortex enables communication between the hippocampus and DMN regions in healthy controls, but not in individuals reporting cognitive decline or diagnosed with prodromal Alzheimer's disease (Dillen et al., 2017). Ratings of ego dissolution under LSD have been shown to correlate with decreased resting-state functional connectivity between the retrosplenial cortex and the parahippocampus (Carhart-Harris et al., 2016b). If the retrosplenial cortex acts as a gateway between the hippocampal formation and specific DMN regions for memory retrieval, the correlation between this change in connectivity and ego dissolution may be related to a loss of access to autobiographical memories. It is also interesting to note that damage to the retrosplenial cortex has been linked with retrograde amnesia (Valenstein et al., 1987; Takayama et al., 1991; Gainotti et al., 1998; Oka et al., 2003), and damage to the hippocampal formation has been associated with both retrograde amnesia (Scoville and Milner, 1957; Squire, 1992; Steinvorth et al., 2005) and loss of general world knowledge (Rempel-Clower et al., 1996; Bayley et al., 2006; Gregory et al., 2014).

In summary, narrative aspects of self-consciousness can be radically altered during a specific conscious episode in two ways: through a temporary cessation of self-referential thought and mental time travel, or more dramatically through a temporary loss of access to semantic autobiographical information, resulting in a complete breakdown of one's personal identity. While the former can seemingly be induced both by contemplative practices and by psychedelic drugs, the latter appears to be more specific to psychedelics, and may be related to more pronounced reductions in DMN integrity—which does not appear to be such a reliable feature of meditation.

In the first case, the retrieval of self-related information is effectively reduced, via attentional control in the case of meditation, and perhaps via an involuntary attentional shift in the case of psychedelic states. In the second case, even the dispositional ability to retrieve such information is temporarily impaired, which amounts to a form of reversible amnesia. It is important to note that these alterations of narrative aspects of self-consciousness come in degrees. At low doses of psychedelics or in novice meditators, self-referential spontaneous thoughts and mental time travel are unlikely to disappear entirely for an extended period of time. By contrast, states of deep absorption achieved by advanced meditators or with higher doses of psychedelics are more likely to involve a complete cessation of these thoughts. Similarly, the disruption of *access* to self-related information may be partial or total in different cases. For example, there is a difference between the inability to remember what one has done the day before, the inability to remember one's name, and the inability to remember anything at all about oneself, including that one belongs to the human species (which has been reported, *post-hoc*, with certain doses of some psychedelics). Thus, narrative aspects of self-consciousness may be temporarily disrupted in different ways and to varying degrees by meditation and psychedelics and it is not yet clear whether these different degrees or grades lie on a linear or nonlinear scale.

### Disruption of multisensory aspects of self-consciousness

While self-related thoughts are a paradigmatic example of self-consciousness, it is widely agreed that self-consciousness is not strictly limited to the cognitive domain. In particular, a number of authors have stressed the need to distinguish between the “narrative self,” congruent with narrative aspects of self-consciousness outlined in the previous section, and the “minimal” or “embodied” self (Damasio, 1999; Gallagher, 2000; Legrand and Ruby, 2009; Christoff et al., 2011; Musholt, 2013; Zahavi, 2014). For example, Gallagher defines the minimal self as “a consciousness of oneself as an immediate subject of experience, unextended in time” (Gallagher, 2000, p. 15), by opposition with the temporal thickness of the narrative self-woven by autobiographical memories and self-projection to the future. Moreover, many authors have insisted on the idea that the minimal self is crucially linked to embodiment and agency, equating this basic form of self-consciousness with an awareness of oneself as an embodied agent (Legrand, 2006; Bayne and Pacherie, 2007; Christoff et al., 2011; Seth, 2013). While the distinction between high-level/narrative and minimal/embodied selfhood is helpful as a first pass to clarify the umbrella notion of self-consciousness, it remains somewhat ambiguous and potentially simplistic as such. As we have seen, the “narrative self” is better construed as a family of distinct self-referential processes, which may or may not involve mental time travel, be spontaneous, or recruit abstract semantic information. Likewise, the “embodied” or “minimal” self may be construed as a complex set of somatosensory and agentive aspects of self-consciousness which can come apart in special cases (Blanke and Metzinger, 2009).

At least three constructs that have been related to a basic form of self-consciousness rooted in multisensory processing may be distinguished, namely: (a) the sense of body ownership, namely the alleged sense of “mineness” that one experiences with respect to one's own body or individual limbs; (b) bodily awareness in general, namely the awareness of any bodily sensation, either internal (interoception and proprioception) or external (tactile); and (c) spatial self-location, namely the experience of being located somewhere in space with respect to one's perceived environment.

#### The sense of body ownership

Body ownership is a controversial notion, as it is not obvious that there is a phenomenology of ownership (a sense of “mineness” over one's body) in daily experience, or how to characterize such phenomenology (Alsmith, 2014; Bermúdez, 2015; De Vignemont, forthcoming). Clinical and experimental evidence suggests that individuals may see body parts or feel tactile sensations originating from them without experiencing them as their own. Indeed, subjects diagnosed with the monothematic delusion known as somatoparaphrenia routinely deny ownership of a limb, despite the fact that nociception and touch may be preserved in the rejected body part (Melzack, 1990; Bottini et al., 2002; see De Vignemont, forthcoming). In the rubber hand illusion, one of the subjects' hands is hidden and stroked synchronously with a rubber hand placed in an anatomically congruent position in front of them (Botvinick and Cohen, 1998); not only do healthy participants report experiencing an illusory ownership over the fake hand, but they also report a loss of ownership over their real hand (Valenzuela Moguillansky et al., 2013). Moreover, a number of physiological measurements appear to indicate that the real limb is temporarily ‘disowned' by the body during the illusion, such as a drop in temperature and an increase in histamine reactivity in the participant's real hand (Moseley et al., 2008; Barnsley et al., 2011; but see Rohde et al., 2013). Beyond individual body parts, questionnaire data suggest that ownership over one's *whole body* can be manipulated in so-called full-body illusions induced by synchronous visuotactile stimulation, during which the participant's body—seen in front of them through a head-mounted display—may no longer feel like their own (Ehrsson, 2007; Blanke and Metzinger, 2009).

Although still a matter of controversy, many authors interpret these data as evidence that ordinary conscious experience involves a sense of body ownership that can both be experimentally manipulated and disrupted in clinical cases. Experiencing one's body as one's own would constitute a form of self-consciousness that does not require the possession or deployment of a self-concept, unlike thinking of oneself. Assuming that such a sense of body ownership is indeed ubiquitous at least in the ordinary experience of neurotypical individuals, is there any evidence that it can be altered by meditation and psychedelics? Interestingly, recent studies by Aviva Berkovich-Ohana and colleagues suggest that mindfulness meditation can indeed induce a loss of body ownership (Dor-Ziderman et al., 2013, 2016; Ataria et al., 2015). Ataria and colleagues studied a single highly experienced meditator “S” (with around 20,000 h of practice) trained in the Satipathana and Theravada Vipassana traditions, who was reportedly able to voluntarily induce a state in which basic aspects of self-consciousness appeared to fade away. In particular, S described the dissolution of the sense of body ownership in a series of open-ended interviews following the methodological principles of the microphenomenological interview technique, designed to elicit fine-grained reports of subjective experience while minimizing potential for confabulation (Petitmengin, 2006; Petitmengin and Lachaux, 2013). S reported that in the altered state of consciousness he achieved through meditation “there really isn't any [sense of ownership]. It's a feeling of dissolving. [It is] hard to distinguish between senses in the body and senses outside [*sic*]… There is no sense of mine [and] there is no sense of me” (Ataria et al., 2015, Supplementary Material). While evidence from single-subject studies should be treated as tentative and interpreted with caution, Dor-Ziderman and colleagues also tested 12 long-term mindfulness meditators in a neurophenomenological study combining magnetoencephalogram (MEG) recording and first-person reports (Dor-Ziderman et al., 2013). Participants were instructed to voluntarily induce a “selfless” mode of awareness characterized as “momentary phenomenal experience free of the sense of agency and ownership” (Dor-Ziderman et al., 2013, p. 3). Subjective reports were analyzed and grouped in categories validated by 12 naïve referees. Four reports were grouped in the category of experiences lacking a sense of ownership, including the following descriptions: “I understood that it was just a sensation, it was not the hand itself… there was a deep thought that all this was not mine” (subject 9); “it was emptiness, as if the self-fell out of the picture. There was an experience but it had no address, it was not attached to a center or subject” (subject 12); “it was to be aware of the body, the sensations, pulse, location of limbs, sounds and sights—to be only a witness to all this” (subject 14) (Dor-Ziderman et al., 2013, p. 6).

There are also many reports of depersonalization-like experiences induced by psychedelics, suggesting that the sense of body ownership can be pharmacologically manipulated (Guttmann and Maclay, 1937; Savage, 1955; Von Mering et al., 1955; Sedman and Kenna, 1964; Studerus et al., 2011). Reports of drug-induced ego dissolution frequently include descriptions of a loss of ownership over one's body (e.g., “I felt disconnected from my physical being, my body”; “[I] looked down at my hand and didn't feel anything that would indicate that this was my hand I was looking at”; R.M., unpublished data from online survey). An intriguing hypothesis is that top-down constraints on body representation are loosened in the psychedelic state. In the rubber hand illusion, for example, it has been hypothesized that the high-level prior probability that one's actual hand is truly one's own is decreased (Limanowski and Blankenburg, 2013; Apps and Tsakiris, 2014). There is preliminary evidence for a weakening of top-down perceptual priors in the psychedelic states, as evidenced by reduced binocular rivalry switching rate and occasional phenomenal fusion of rival images under psilocybin and ayahuasca (Frecska et al., 2003; Carter et al., 2007), as well as reduced susceptibility to the “hollow mask” illusion (unpublished result of Torsten Passie's hollow mask study at the Hannover Medical School in Germany). In addition, a recent study showed that LSD attenuates top-down suppression of prediction error in response to surprising auditory stimuli, as measured by mismatch negativity in the auditory oddball paradigm (Timmermann et al., in press). It is possible that a similar disruption of top-down processing of somatosensory stimuli plays a role in the modulation of body ownership by psychedelics (see also Swanson, 2018 for a discussion of the effects of psychedelics on top-down processing).

Thus, assuming that there is a phenomenology of body ownership in ordinary experience, available evidence from open-ended interviews and self-report questionnaires tentatively suggests that it can go missing during certain conscious states induced by meditation and psychedelics—not only for specific body parts but also for the whole body. However, more evidence is needed to confirm this hypothesis, as well as a rigorous definition and measurement of the sense of body ownership.

#### Bodily awareness

Bodily awareness can be defined as the conscious awareness of bodily sensations in general, including tactile, proprioceptive and interoceptive stimuli. A number of authors have argued that bodily awareness constitutes a form of self-consciousness, in part because it grounds self-ascriptions that are immune to error through misidentification (Bermúdez, 2011; De Vignemont, 2011). When one feels pain, for example, one cannot be wrong about *who* is in pain; generally, bodily sensations are said to be immune to error through misidentification insofar as the only body that one can have access to through them is one's own. This kind of consideration has led some philosophers to argue that bodily awareness constitutes a basic form of self-consciousness through which an embodied subject is directly conscious of the bodily self (Evans, 1982; Cassam, 1997; Bermúdez, 2011). In recent years, a similar idea has emerged within neuroscience with a particular focus on interoception, the awareness of internal bodily sensations such as cardiac, respiratory and gastric signals. It has been argued that interoceptive awareness grounds a core sense of self in normal experience, anchoring oneself in one's body (Damasio, 1999; Craig, 2002; Seth, 2013; Tsakiris, 2017). It should be noted that this idea does not necessarily rest on the hypothesis that there is a specific phenomenology of body ownership, although it is not always easy to disentangle the claim that one's body is experienced as one's own from the claim that one's bodily sensations underlie a consciousness of oneself as a bodily subject. The distinction of these two claims turns on the clarification of their theoretical commitments. Here, we will leave this issue aside, and assume that bodily awareness constitutes a basic form of self-consciousness, whether or not it is associated with a specific phenomenology of ownership.

Can meditative practices and psychedelics induce a partial or complete loss of bodily awareness? Given the focus of many meditation practices on bodily sensations, either through open awareness of all present moment sensations (in open monitoring) or through focal awareness of the breath (in focused attention), one would not expect bodily awareness to fade away during meditation practice. The meta-analytic cluster of activation of the insular cortex during OM, FA, and LK reported in the previous section (Fox et al., 2016) also suggests that bodily awareness is central to these meditation practices, given that the insula is an important hub for the processing of interoceptive signals (Critchley et al., 2004; Craig, 2009; Simmons et al., 2013). Nonetheless, there is preliminary evidence that bodily awareness can be reduced in certain forms of mindfulness meditation, at least for highly experienced practitioners. For example, subject S from Ataria and colleagues' study reported that in the “selfless” altered state of consciousness voluntarily achieved through meditation, bodily sensations were “almost invisible” and reduced to a subtle and indistinct background presence: “there is a sense of something happening, it is very hard to tell if there is a sense of body, it is more in the background… a sense of body-ness, but it's so spread… I am not dead; there is a kind of very light sense of body in this experience” (Ataria et al., 2015, Supplementary Material). In their analysis of the same data, Dor-Ziderman and colleagues comment that “even when the [sense of boundaries] disappears, a minimal level of dynamic proprioception continues to exist: there remains a sense that there is a body without any experience of [a sense of boundaries]” (Dor-Ziderman et al., 2016, p. 3).

It is debatable whether proprioceptive awareness was retained in this state, given the difficulty that subject S had in locating his bodily sensations on a body-part-centered spatial frame of reference (“I can't tell you at all which [hand] is right and which is left. I don't know”, Ataria et al., 2015, Supplementary Material). Nonetheless, it is plausible that some degree of *interoceptive* awareness was preserved, which is consistent with the fact that the body was merely experienced as some kind of vague background presence. Berkovich-Ohana and colleagues also found that experienced mindfulness meditators could significantly inhibit awareness of bodily sensations during their practice (Berkovich-Ohana et al., 2013): “The experience of the body faded. There was a sense of body in the background, not in front of consciousness” (subject 4); “A wide experience with un-defined boundaries. A sense… that I am dissolving, my body dissolving.” (subject 5); “There was a vanishing of bodily sensations” (subject 11).

While it remains unclear whether bodily awareness can completely disappear during meditation, there is evidence that subjects can lack any awareness of their body and of specific bodily sensations in altered states induced by psychedelic substances. As previously mentioned, early studies with mescaline and LSD reported depersonalization-like effects which occasionally involved a loss of bodily awareness (Guttmann and Maclay, 1937; Savage, 1955; Von Mering et al., 1955; Sedman and Kenna, 1964). In the psychometrically validated 5D-OAV questionnaire commonly used to assess the subjective experience of altered states of consciousness (Studerus et al., 2010), healthy participants score rather high on the “disembodiment” factor, which includes the item “it seemed to me as if I did not have a body anymore,” after administration of psilocybin (Kometer et al., 2012; Bernasconi et al., 2014; Preller et al., 2016; Pokorny et al., 2017) and LSD (Schmid et al., 2015; Carhart-Harris et al., 2016a,b; Kraehenmann et al., 2017; Liechti et al., 2017; Preller et al., 2017b). The strength of the subjective effects of LSD (as measured by the mean score on the 5D-ASC questionnaire) was found to correlate with the magnitude of increase in functional connectivity in a somatomotor network including the primary motor and sensorimotor cortices, the caudal premotor cortex and the superior parietal lobule (Preller et al., 2017a).

In a recent neuroimaging study of DMT, a drug whose short lasting subjective effects are more intense and immersive than those of psilocybin or LSD, almost all participants reported a loss of awareness of their body for several minutes during the peak of the experience in *post-hoc* microphenomenological interviews: “I lost awareness of gravity and awareness of my body” (Timmermann et al., in preparation). There is also considerable anecdotal evidence from narrative reports that psychedelics can radically disrupt bodily awareness, an effect often associated with the dissolution of the sense of self (Millière, 2017): “It felt as if ‘I' did no longer exist. There was purely my sensory perception of my environment, but sensory input was not translated into needs, feelings, or acting by “me”. Also, I felt disconnected from my physical being, my body” (R.M., unpublished data from online survey). Anecdotal evidence regarding the dramatic effects of 5-MeO-DMT suggests that this drug may be particularly effective at suppressing bodily awareness, although controlled studies of these effects are needed^5^.

Thus, it seems that certain meditation practices may inhibit awareness of bodily sensations, reduced in some cases to a mere background interoceptive awareness, while psychedelic substances (DMT and 5-MeO-DMT in particular) may completely suppress awareness of the body. Sensory deprivation is likely to be a factor in this reduction of bodily awareness: meditators are usually sitting in silence with eyes closed, while participants of neuroimaging studies of psychedelics are often lying down in supine position with a blindfold. The lack of somatosensory and motor feedback in these states may play an important role in the loss of bodily awareness. Interestingly, two recent findings emphasize the influence of sensory deprivation on the plasticity of body representation: short-term visual deprivation has been shown to lead to significantly larger proprioceptive drift in the rubber hand illusion (Radziun and Ehrsson, 2018), while audio-visual sensory deprivation has been found to degrade the boundary of the whole body peripersonal space (Noel et al., 2018). These results suggest that sensory deprivation enhances the flexiblity of body representation, and could facilitate the disruption of bodily awareness in meditation and psychedelic states.

Finally, it is important to note that psychedelic drugs, just like some styles of meditation, may also *increase* awareness of the body, especially at lower doses associated with salient and unusual bodily sensations (see section Toward a multidimensional model of altered self-consciousness below). Finally, it can also be observed that drug-induced states are not the only known altered states of consciousness in which bodily awareness can fade away altogether; there is good evidence that this can also be the case in so-called “bodiless” dreams (LaBerge and DeGracia, 2000; Cicogna and Bosinelli, 2001; see Windt, 2010, 2015; Occhionero and Cicogna, 2011) and “asomatic” out-of-body experiences (Alvarado, 2000; see Metzinger, 2013).

#### Spatial self-location

A third notion associated with a minimal form of self-consciousness rooted in multisensory integration is spatial self-location (Blanke and Metzinger, 2009; Serino et al., 2013; Maselli, 2015). It has been argued that perceptual experience, and visual experience in particular, is *self-locating* (Cassam, 1997; Noë, 2005; Schwenkler, 2014). This idea typically combines two claims: first, in virtue of being structured by an egocentric frame of reference^6^, visual experience represents the location of the point of origin of this reference frame relatively to environmental landmarks; secondly, the location of this point of origin is represented *as* the location of the subject (i.e., where *I* am located with respect to objects in the visual scene). This analysis may be extended to other sensory modalities whose content presumably has a perspectival structure centered onto a single point of origin, such as auditory perception (Coleman, 1963; Zahorik et al., 2005; Kolarik et al., 2016).

Whether perspectival structure always entails *self-locating* content—namely a representation of the location of the origin as the location of the *subject*—is a matter of debate (Alsmith, 2017). Some authors have argued that self-locating content also requires the ability to act upon objects perceived in one's environment (Brewer, 1992; Schellenberg, 2007; Alsmith, 2017). Nonetheless, it is generally agreed that ordinary perceptual experience has self-locating content. In other words, one normally experiences oneself as located at a certain distance with respect to objects perceived in one's environment. Interestingly, both questionnaire data and behavioral measurements suggest that self-location can be manipulated in full-body illusions, such that subjects may feel located outside of their own body (Ehrsson, 2007; Guterstam et al., 2015) or closer to the virtual avatar over which they feel ownership during the illusion (Lenggenhager et al., 2007, 2009; Ionta et al., 2011). Similarly, out-of-body experiences and heautoscopic phenomena of clinical origin, in which subjects hallucinate seeing their own body from the outside, appear to involve a shift of self-location (often to an elevated position) associated with a hallucinatory visuospatial perspective (Blanke and Arzy, 2005; Blanke and Mohr, 2005).

Such experimental and clinical evidence isolating spatial self-location as a dissociable component of ordinary experience has led researchers to claim that it constitutes an important aspect of bodily self-consciousness (Blanke and Metzinger, 2009; Blanke et al., 2015), and even a minimally sufficient condition for self-consciousness (Metzinger, 2013; Windt, 2015). Interestingly, there is evidence that both meditation and psychedelics may radically disrupt the experience of being located somewhere in space; moreover, this disruption appears to be often associated with reports of “selflessness” or “ego dissolution,” suggesting that spatial self-location is indeed a basic and important building block of self-consciousness. The highly experienced mindfulness meditator studied by Ataria and colleagues described the selfless state he reportedly achieved for the experiment in the following terms: “it's like falling into empty space… and a sense of dissolving… and there really isn't a center… I don't have any kind of sense of location… I have no idea where I am in stage three, it's all background, I'm not there basically, just world, so there's no real location at all in stage three. It's very minimal, almost nothing… When there's no boundary, there's no personal point of view, it's the world point of view, it's like the world looking, not [me] looking, the world is looking.” (Ataria et al., 2015, Supplementary Material). As Dor-Ziderman and colleagues comment, in this meditation-induced altered state of consciousness, it seems that “the sense of orientation in space is lost altogether” (Dor-Ziderman et al., 2016, p. 3). In another neuroimaging study of long-term mindfulness meditation practitioners, Berkovich-Ohana and colleagues found that some of them were able to induce what they described as a state of “spacelessness,” reported in the following terms: “the center of space became endless, without a reference point in the middle” (subject 4); “it was a sense of spaciousness, boundlessness… there was no clarity where the center is and where is the periphery. There was no quality of border” (subject 11) (Berkovich-Ohana et al., 2013).

Likewise, there is converging evidence from subjective reports that psychedelic drugs can induce a loss of spatial self-location, associated to loss of boundary between self and world and a feeling of unity with everything (Millière, 2017): “I felt myself mold into the world around me…,” “My mind started to blend with everything” (R.M., unpublished data from online survey). On the psychometrically-validated 5D-ASC questionnaire, subjects score high on the factor related to “experience of unity” after administration of LSD (Schmid et al., 2015; Carhart-Harris et al., 2016a,b; Kraehenmann et al., 2017; Liechti et al., 2017; Preller et al., 2017b) and to a slightly lesser extent psilocybin (Kometer et al., 2012; Bernasconi et al., 2014; Preller et al., 2016; Pokorny et al., 2017). This factor includes the items “it seemed to me that my environment and I were one” and “everything seemed to unify into oneness”. Moreover, studies using custom questionnaire items have also measured high scores for “I experienced a sense of merging with my surroundings” after administration of psilocybin and LSD (Muthukumaraswamy et al., 2013; Carhart-Harris et al., 2016b; Schartner et al., 2017). A recent online survey on the effects of 5-MeO-DMT with 515 participants also found that the overwhelming majority of respondents scored positively on items of the Mystical Experience Questionnaire corresponding to “Loss of your usual sense of space” (95% of respondents), “Loss of usual awareness of where you were” (88% of respondents) and “Being in a realm with no space boundaries” (87% of respondents) (Davis et al., 2018).

In addition to questionnaire data from controlled studies, there is a large amount of anecdotal evidence from online narrative reports that DMT and 5-MeO-DMT can induce a loss of self-location: “at the time I didn't know where it was, or where I was…I didn't know what had happened before this point, in-between, sideways, up, down or anywhere” (DMT, report from www.dmt-nexus.me), “I was the universe, I was everywhere and nowhere, everything and nothing all at the same time” (5-MeO-DMT, report #49690 from Erowid.org), “The feeling was very cosmic, of oneness with everything” (5-MeO-DMT, report #78485 from Erowid.org), “an immediate complete dissolution of any identity and a merging into the Oneness, timeless, pure awareness and light energy of the Universe… Similar in some ways with a previous *Samadhi* meditation experience” (5-MeO-DMT, report #5804 from Erowid.org; see below on *Samadhi* meditation). A similar phenomenological report can be found in a (non-academic) monograph on 5-MeO-DMT: “I was completely disconnected somatically, unable to locate or feel my body… unable to locate myself—or anything else—anywhere in particular. I had no body, not even the slightest semblance of a dream-body or mental-body, and I had absolutely no sense of where I was” (Masters, 2005). Taken at face value, these reports suggest that psychedelic drugs can induce experiences which may have perceptual content without spatial—or at least self-locating—content. Although narrative reports from online databases offer at best anecdotal evidence, their convergence with questionnaire data from multiple controlled studies suggests that they may be taken into consideration to inform hypotheses about the effect of psychedelics on the sense of self-location.

We note that these reports of meditative and psychedelic experiences lacking spatial self-locating content might be reminiscent of reports of conscious episodes during dreamless sleep, which allegedly lack any form of self-consciousness and spatial content (Thompson, 2014a; Windt et al., 2016). Jennifer Windt has argued that such dreamless sleep experiences might be characterized by “pure subjective temporality,” conceived as the minimal phenomenology of temporal self-location (“nowness”) and duration (Windt, 2014). If such states do exist, we can plausibly hypothesize that they might also be induced by meditation and psychedelic drugs, on the basis of the preliminary evidence discussed in this section. However, we also note that many reports of drug-induced ego dissolution, especially with 5-MeO-DMT, insist on the *timeless* character of the experience, described as a complete loss of the sense of temporal duration. The aforementioned online survey on the effects of 5-MeO-DMT found that the overwhelming majority of respondents scored positively on items of the Mystical Experience Questionnaire corresponding to “Loss of your usual sense of time” (97% of respondents), “Experience of timelessness” (90% of respondents), “Sense of being outside of time, beyond past and future” (89% of respondents) and “Feeling that you experienced eternity or infinity” (88% of respondents) (Davis et al., 2018). Interestingly, some mindfulness meditators have been reported to achieve a similar disruption of the phenomenology of duration through their practice (Berkovich-Ohana et al., 2013). Thus, we tentatively suggest that some drug-induced and meditative states might lack both *spatial* and *temporal* self-locating content.

From a neurophysiological point of view, it is interesting to note that the intensity of drug-induced ego dissolution reported by participants under LSD was found to correlate with the magnitude of increased functional connectivity in the bilateral insular cortex and the temporoparietal junction (Tagliazucchi et al., 2016). Meanwhile, Dor-Ziderman and colleagues found that the transition from minimal self-consciousness to a “selfless” state characterized by a loss of body ownership and self-location in experienced meditators was correlated with a decrease in beta power in the temporoparietal junction (Dor-Ziderman et al., 2016). These findings are intriguing since there is evidence from neuroimaging studies of out-of-body experiences and full-body illusions that the temporoparietal junction plays a role in processing spatial self-location (Blanke and Arzy, 2005; Ionta et al., 2011; Blanke, 2012). Given that a number of intracranial EEG and MEG studies have suggested that beta oscillations encode top-down modulations of predictions (Bauer et al., 2014; Michalareas et al., 2016; Sedley et al., 2016), it is intriguing to speculate that decreased beta power in the TPJ may relate to weakened top-down constraint on multisensory processing underlying self-location. In addition, the insular cortex is associated with the integration of interoceptive information (Craig, 2009; Simmons et al., 2013), and may play an important role in body awareness and ownership (Aspell et al., 2013; Seth, 2013; Suzuki et al., 2013). Thus, while the modulation of beta oscillatory power and functional connectivity in the temporoparietal junction in some psychedelic and meditative states may be linked to the disruption of spatial self-location, the modulation of functional connectivity in the insula during drug-induced ego dissolution might be specifically related to the loss of bodily awareness. However, more data is needed to confirm these hypotheses.

To conclude this section, it should be noted that our overview of narrative and multisensory aspects of self-consciousness has left out the notion of the sense of agency, typically defined as the experience of being in control of one's actions (Haggard, 2017). There are three reasons for this omission aside from spatial constraints. Firstly, the sense of agency is an ambiguous concept, which has been construed both as a feeling of control over one's bodily movements allegedly produced by comparator mechanisms, and as a feeling of control over the production of one's thoughts. Secondly, there is little available evidence regarding how the sense of agency, in either construal, may be modulated by meditation and drug-induced states. Although experiences of self-loss with both psychedelics and meditation typically occur when subjects are immobile (in seated or supine position), increased voluntary control over one's breathing in certain styles of meditation could modulate the sense of bodily agency. Further evidence could be provided by measures of intentional binding during meditation and psychedelic states. Likewise, one could expect attentional control of spontaneous thoughts in meditation to have an effect on the sense of cognitive agency, if this notion is valid. Finally, it is worth mentioning that the claim that voluntary movements and thoughts are ordinarily accompanied by a pervasive sense of agency has recently come under criticism (Grünbaum, 2015; Grünbaum and Christensen, 2017; Parrott, 2017). According to a more deflationary account, there is no special phenomenology of agency in ordinary experience^7^.

## Pure consciousness, non-dual awareness and total selflessness

In this section, we examine in more detail some meditation practices which explicitly aim at a dissolution of all aspects of self-consciousness, and consider how the resulting global states of consciousness compare to drug-induced states. More specifically, we focus on practices targeting the subject-object dichotomy which allegedly structures ordinary conscious experience, in order to reach a state of “non-dual awareness” (NDA; Josipovic, 2010), or even induce a state of consciousness supposedly empty, i.e., devoid of any content (“pure consciousness”). It is an open question whether NDA differs from pure consciousness, and in what respect; but a critical examination of descriptions of these states suggests that both may involve a dramatic form of self-loss characterized by a dramatic inhibition of the dimensions of self-consciousness outlined in the previous section.

### Non-dual awareness

Non-dual awareness meditation (NDA) refers to a family of practices which can be found in several Eastern contemplative traditions, including Dzoghen and Mahāmudrā within Tibetan Buddhism, and Advaita Vedanta and Kashimiri Shaivism within Hinduism (Josipovic, 2010; Dunne, 2011). NDA meditation rests on three core assumptions: (a) ordinary experience is “dual” or dichotomous, insofar as it is structured around a subject-pole and an object-pole; (b) this subject-object dichotomy is illusory, because conscious awareness as such is not fundamentally dual; (c) it is possible, through the practice of NDA meditation, to dispel this illusion and directly experience conscious awareness as non-dual. All of these assumptions are worth discussing. The first assumption, in particular, requires clarification. It is rather uncontroversial that all conscious mental states have a subject of experience, insofar as “an experience is impossible without an experience” (Frege, 1956, p. 299, translation modified). This is merely a metaphysical requirement of conscious experience^8^. However, assumption (a) goes further in claiming that the phenomenal character of conscious experience is itself structured by a subject-object dichotomy.

There are at least two ways to understand this claim. As we have seen in the previous section, a number of components of ordinary experience can be related to a form of self-consciousness—including self-related thought, body ownership, bodily awareness and spatial awareness. One can claim that conscious experience normally involves a background awareness of oneself which is reducible to one or several of these components (Damasio, 1999; Blanke and Metzinger, 2009; Bermúdez, 2011; Seth, 2013). According to this reductionist interpretation, ordinary consciousness is structured by a subject/object dichotomy insofar as we are normally aware of ourselves via thought, perception and bodily sensations in addition to being aware of external objects. A second interpretation holds that there is a form of *sui generis* self-awareness in experience which is irreducible to the cognitive, bodily and spatial features of experience: “being presented with something necessarily involves being pre-reflectively and pre-conceptually aware of being the subject to whom something is presented” (Nida-Rümelin, 2017, p. 66; see also Strawson, 1999; Kriegel, 2009; Zahavi, 2014; Guillot, 2017). According to this second interpretation, the seemingly dichotomous nature of experience does not rest on a specific kind of self-representing content, but on the very nature of conscious representation in general, which is structured by an implicit distinction between the represented objects and the subject to whom those objects are presented. In similar fashion, Evan Thompson has argued that ordinary experience is infused with a sense of mineness such that every thought, emotion, perception or sensation is experienced *as one's own*, and has also suggested that this feature can disappear during meditation (Thompson, 2014b, p. 362).

On both of these interpretations, conscious experience is structured by a subject-object dichotomy insofar as it involves an awareness of oneself in addition to the awareness of external objects. Accordingly, non-dual awareness states can be construed as conscious states which lack the background self-awareness normally present in experience. However, a core assumption of non-dual awareness meditation practices is that the subject-object dichotomy supposedly found in ordinary experience is illusory, in line with the so-called “no self” doctrine of Buddhism (*anātman* in Sanskrit or *anattā* in Pali). Thus, non-dual awareness practice is supposed to reveal that the putative phenomenological distinction between oneself and one's experience of the external world is ultimately an illusion^9^.

While this general idea appears to be consistent with recent proposals regarding the notion of non-dual awareness, it is not always clear which of the two interpretations outlined above is favored. For example, Wolfgang Fasching has argued that in normal experience, subjects are aware of their body and location in addition to objects of the external world, which suggests that he favors the reductionist interpretation of the subject/object dichotomy. In the same vein, he suggests that self-consciousness is rooted in the identification of oneself with “certain configurations of experienced contents as opposed to others” (Fasching, 2008, p. 476), which is also consistent with the first interpretation. However, he goes on to argue that “in perception I am necessarily co-conscious of myself” (Fasching, 2008, p. 472), because the subject/object polarity is built in all conscious representational states. He further claims that some meditation practices can reveal that “I am not something ‘inner' as distinct from external objects” and that “there is no ‘I' to which things are given, there is just the event of givenness” (Fasching, 2008, p. 478). In this context, meditation is conceived as a way of becoming aware of consciousness as such, without the illusory distinction between the subject and the objects of experience. Fasching cites a central text of the Vedic tradition, the Māṇḍūya Upaniṣad, which describes the state of non-dual awareness reached through meditation as one in which the subject is “not conscious of the internal world, nor conscious of the external world” (Deutsch, 1969, p. 62). Similarly drawing on the Vedic tradition, Miri Albahari suggests that non-dual awareness meditation involves “a direct realization that consciousness… is ownerless” (Albahari, 2010, p. 104).

These descriptions seem consistent with the second interpretation above: in ordinary conscious states, subjects have a sense that all of their experiences are *theirs, given to them*, or *owned by them*; in turn, this introduces an artificial distinction between oneself as the subject of experience and the experiences themselves; finally, trained meditators can dispel this illusion by becoming aware of consciousness itself as a non-dual process. Although we cannot discuss this proposal at length within the scope of this article, it is worth underlining that it rests on a controversial picture of ordinary experience. Indeed, the idea that consciousness is normally infused with a special sense of phenomenal mineness or awareness of oneself as the owner of one's experiences is far from obvious and has been met with a number of objections (see Howell and Thompson, 2017; O'Conaill, 2017; Chadha, 2018; McClelland, forthcoming; Wu, forthcoming).

By contrast, the first interpretation of non-duality outlined above seems consistent with the evidence presented in the previous section: the inhibition of self-related thoughts, body ownership, bodily awareness and self-location should entail a blurring or dissolution of the boundary between self and world, and the associated “unitive experience”—identified by Walter Stace as the core feature of so-called “mystical-type experiences” (Stace, 1960). Although narrative and multisensory aspects of self-consciousness are not illusory as components of ordinary experience, their transient cessation in meditation or drug-induced states may be interpreted as revealing that their association with a more substantial notion of selfhood is fallacious (see also Metzinger, 2003; MacKenzie, 2016; Letheby and Gerrans, 2017). It is worth noting, however, that classic accounts of non-dual awareness in the Buddhist and Hindu traditions emphasize its reliance on the loss of narrative aspects of self-consciousness more than the loss of multisensory aspects. Non-dual Mahāmudrā practice, for example, instructs students to “drop thoughts of past, present and future and release the mind into its natural state of clear, non-conceptual awareness” (Dunne, 2011, p. 81). Consequently, it is possible that global states of consciousness reached through NDA meditation may preserve some awareness of bodily sensations^10^.

In summary, we have suggested that the notion of NDA states can be understood in two different ways. According to the first interpretation, they are conscious states in which both narrative and perhaps multisensory aspects of self-consciousness are radically disrupted, such the distinction between oneself and the external world no longer has an experiential basis. According to the second interpretation, ordinary conscious states involve a minimal form of self-awareness which is not tied to any specific content, but to a special sense of “self-givenness” or “mineness” built in normal experience; in NDA states, this feature supposedly goes missing, such that subjects become aware of consciousness itself as an ownerless process. While the second interpretation rests on a controversial assumption about normal experience, the first is consistent with the evidence presented so far regarding the phenomenology of alleged “selfless” states in meditation and drug-induced states^11^.

Although there have been few neuroimaging studies of NDA meditation, the available evidence is intriguing. In the resting state, activity in the default-mode network has been found to be negatively correlated with activity in a set of regions commonly recruited in attention-demanding tasks, in particular the sensory (Golland et al., 2007), dorsal attention network and fronto-parietal control network (Greicius et al., 2003; Fox et al., 2005; Uddin et al., 2009; Chai et al., 2012; Carbonell et al., 2014). A similar pattern of anticorrelation has been reported during mindfulness meditation (Brewer et al., 2011; Kilpatrick et al., 2011; Hasenkamp et al., 2012), and there is some preliminary evidence of increased anti-correlation between the DMN and a task-positive network during focused attention meditation compared to the resting state (Josipovic et al., 2012). However, Josipovic and colleagues found that this anticorrelation was significantly *decreased* during NDA meditation compared to both focused awareness meditation and the resting state (Josipovic et al., 2012; Josipovic, 2014). It is worth noting that a similar pattern of increased correlation between the DMN and the habitually anti-correlated networks has been observed during various other forms of meditation, such as mantra recitation (Berkovich-Ohana et al., 2015) as well as choiceless-awareness, loving-kindness and concentration in experienced meditators compared to novice meditators (Brewer et al., 2011). Thus, it is questionable that this change of connectivity is unique to NDA meditation. Interestingly, a similar decrease of the anticorrelation between the DMN and a task-positive network has been observed after administration of psilocybin, and was hypothesized to correlate with decreased separatedness between internally and externally focused states (Carhart-Harris et al., 2013). Although this hypothesis is speculative, it is possible that conscious states induced through NDA meditation have a lot in common with certain states of “ego dissolution” induced by psychedelics from a phenomenological point of view. Before discussing this potential convergence, we will assess how NDA may relate to so-called “pure consciousness.”

### Pure consciousness

The notion of pure consciousness seems to originate in Stace (1960, p. 86), who applies it to mystical experiences. A purely conscious state is characterized by “an ‘emptying out' by a subject of all experiential content and phenomenological qualities, including concepts, thoughts, sense perception, and sensuous images” (Gellman, 2017). States which have been qualified as purely conscious include experiences reached through certain forms of meditation (Nash and Newberg, 2013) and mystical experiences (Stace, 1960; Shear, 1994).

There has been considerable debate about whether purely conscious states are even possible. Some have argued that they are impossible because one cannot be conscious without being conscious of anything at all (Bayne, 2007, p. 16) or because one cannot have episodic memory of a state devoid of experiential content (Bagger, 1999, p. 102; Gennaro, 2008). Others have argued that pure consciousness is not ruled out by logical or phenomenological considerations (Woodhouse, 1990; Shear, 1994), and that we might even find empirical evidence of pure consciousness in contemplative practices (Koch, 2004; Baars, 2013; Metzinger, 2013; Nash and Newberg, 2013) or dreamless sleep (Windt et al., 2016). Note that this apparent disagreement could be a mere verbal dispute, because there is an important ambiguity in the notion of a conscious mental state devoid of any content. Indeed, “content” might refer here to *representational* or *phenomenal* content.

Although controversial, the hypothesis that some conscious mental states lack *representational* content has been defended on the ground that states such as moods, pain or orgasm which do not seem to represent anything (Rey, 1998; Kind, 2007; Aydede, 2009). This is in fact the basis of a prominent objection to representationalism, the thesis that all phenomenally conscious states have representational content, or in its stronger version that the phenomenal character of all conscious mental states is wholly constituted by their representational content. But representational contentlessness is presumably not what proponents of pure consciousness have in mind, as they suggest that purely conscious states lack “all determinate phenomenological contents…whatsoever” (Shear, 1994, p. 320). Thus, the strict definition of a purely conscious state would be a conscious mental state lacking phenomenal character. However, this definition cannot be taken literally if we are talking about phenomenal consciousness: by definition, a phenomenally conscious mental state is a mental state such that there is something it like for a creature to be in it (Nagel, 1974), and if there is something it is like to be in a mental state then the mental state has phenomenal character. Therefore, the notion of a (phenomenally) conscious mental state literally lacking phenomenal character is absurd (Gennaro, 2008; Strawson, 2013): the experience of absence is not equivalent to the absence of experience.

There is, however, a more plausible, non-literal definition of pure consciousness as a conscious state lacking *ordinary* phenomenal content (Gennaro, 2008; Strawson, 2013). The question, then, is what counts as an *ordinary* phenomenal content, and how phenomenally “bare” or “sparse” purely conscious states can get. Presumably, if there are phenomenal properties pertaining to self-consciousness in ordinary experience (i.e., the narrative and somatosensory aspects of self-consciousness previously discussed), then such ordinary phenomenal properties should be missing in purely conscious states, among other ordinary phenomenal properties. Defined in this way, pure consciousness appears to be at least conceivable, and constitutes a plausible candidate for a wholly “selfless” state, lacking self-referential thoughts, bodily ownership, body awareness and self-location. The question is now whether purely conscious states can actually occur, specifically during meditation or after psychedelic intake, and whether they differ from states of alleged “non-dual awareness.”

In the Hindu and Buddhist traditions, the meditative practice called *Samadhi* aims at inducing a state of deep absorption (Shankman, 2008). The practice of *Samadhi* includes four stages or *jhanas*, culminating in a “formless” or “objectless” state. It relies on successive shifts of attention, first from the breath to somatosensory pleasure, then to mere positive affect, until all intentional objects have been stripped away and only “one-pointed” awareness remains. In the final stage of *Samadhi* practice (i.e., the last *jhana*), “there is no directing of attention, no representation held in focal attention, no pleasure and no aversion, affective contentment has dropped away, and all that remains is an alert, clear, one-pointed, equanimous awareness.” (Yamashiro, 2015, p. 7).

The aim of this practice is frequently described as reaching a state of pure consciousness which involves a complete loss of self-consciousness: “attaining *Samadhi* is to reach the silent state of pure consciousness where there is no phenomenological content and a loss of any sense of individual self or duality” (Wahbeh et al., 2018, p. 6)^12^. Miri Albahari has suggested that the state of pure consciousness allegedly reached through *Samadhi* meditation could provide evidence for the existence of what she calls “witness-consciousness,” which she defines as the neutral common denominator between all conscious experience (Albahari, 2009). She argues that witness-consciousness has its own intrinsic phenomenal character, which explains why purely conscious states in *Samadhi* are not devoid of phenomenal content, and thus not logically inconsistent. However, this hypothesis seems unduly inflationary: rather than postulating that a special “objectless” phenomenology is ubiquitous in consciousness and is the only thing remaining in purely conscious states, one may seek to describe such states as a form of deep absorption associated with extreme sensory gating which does not have much in common with ordinary experience.

Unfortunately, the neurophysiological evidence regarding *Samadhi* practice is still extremely sparse (Yamashiro, 2015). A single-participant study found decreased activity in Brodmann areas 5 and 7, which may be associated with the representation of the body's orientation in three-dimensional space (Hagerty et al., 2013). This reflected the participant's experience of losing body boundaries. Nash and Newberg (2013) also suggest that activity of the posterior parietal lobule is modulated by *Samadhi*, and “might be critical for distinguishing between the self and the external world” (Nash and Newberg, 2013, p. 8). Another contemplative tradition which aims at inducing pure consciousness is transcendental meditation (TM), whose goal is the “loss of boundaries of time, space, and body sense that defines the framework for typical waking experience,” but neurophysiological data on TM is also very limited (Travis and Pearson, 2000; Wahbeh et al., 2018).

It is unclear to what extent the notions of non-dual awareness and pure consciousness should be distinguished, both in terms of their conceptualization in the relevant literature on contemplative traditions, and in terms of the phenomenology and neurophysiology of the associated states induced by meditation. According to Josipovic, NDA meditation is not content-driven like focused attention and open monitoring, because it seeks awareness of the background of experience rather than any specific bodily or perceptual content (Josipovic, 2014). Moreover, he argues that NDA meditation also differs from “objectless” meditation “in which the mind is emptied of content and held in an empty state through the force of concentration” (Josipovic, 2014, p. 12)^13^. Indeed, Josipovic contends that pure consciousness practice like *Samadhi* aims at actively “eliminating” each pole of the subject/object dichotomy, while NDA meditation involves the recognition of a background non-dual awareness that “precedes conceptualization and intentionality” (Josipovic, 2014, p. 12). Likewise, John Dunne argues that non-dual awareness differs from objectless meditation techniques such as *Samadhi* insofar as it should not involve any cognitive effort (Dunne, 2011). However, these conceptual distinctions are not straightforward, and there is not yet enough data on the phenomenology and neurobiology of alleged states of “pure consciousness” and “non-dual awareness” to determine whether these are valid and distinct constructs.

As we have suggested, one possible construal of non-dual awareness is in terms of the inhibition of both narrative and multisensory aspects of self-consciousness, leading to a temporary loss of experiential boundary between self and world, or between endogenous and exogenous stimuli. In turn, purely conscious states could be defined as states of extreme absorption involving high sensory gating, whose sparse phenomenal content has little overlap with the rich phenomenology of ordinary wakeful experience. If these tentative definitions are sound, states of pure consciousness should also be states of non-dual awareness, because they should lack ordinary phenomenal content *including* the narrative and multisensory components of self-consciousness. By contrast, non-dual awareness might not entail pure consciousness, because states of total self-loss (lacking narrative and multisensory self-consciousness) need not have very sparse phenomenal content.

This raises the question of whether psychedelic drugs can induce virtually “contentless” states similar to the descriptions of pure consciousness from mystical and contemplative traditions. While most reports of drug-induced ego dissolution involve a rich sensory phenomenology, including vivid hallucinations, this is not always the case. In particular, there is anecdotal evidence that certain compounds such as 5-MeO-DMT may induce states of radical absorption reminiscent of *Samadhi* practice^14^. Indeed, users of 5-MeO-DMT frequently describe an experience of “emptiness,” “nothingness” or “void” which is associated with a cessation of thoughts, extreme sensory deprivation and a complete loss of self-consciousness: “my brain was not conveying anything meaningful from what my senses were receiving; I was completely unaware of my body, experiencing profound stillness” (report #99920 from Erowid.org); “I felt that there was nothing to me and there was nothing around me” (#11701); “the reality around me disintegrated into nothing. I fell into a void [that] I can't even describe” (#23487); “I wasn't anything anymore. I had been broken down into nothingness, into oblivion” (#87426); “my thoughts ceased to exist, and my senses shut off completely. I could not hear, see, smell, taste or feel anything” (#37301). This suggests that states of drug-induced ego dissolution may vary not only according to the extent to which narrative and multisensory aspects of self-consciousness are inhibited, but also according to the richness or sparsity of their phenomenology. While some forms of self-loss induced by psychedelics may involve a rich sensory phenomenology, others are more similar to descriptions of “pure consciousness,” and may be almost devoid of sensory content.

## Toward a multidimensional model of altered self-consciousness

**Figure 1:**
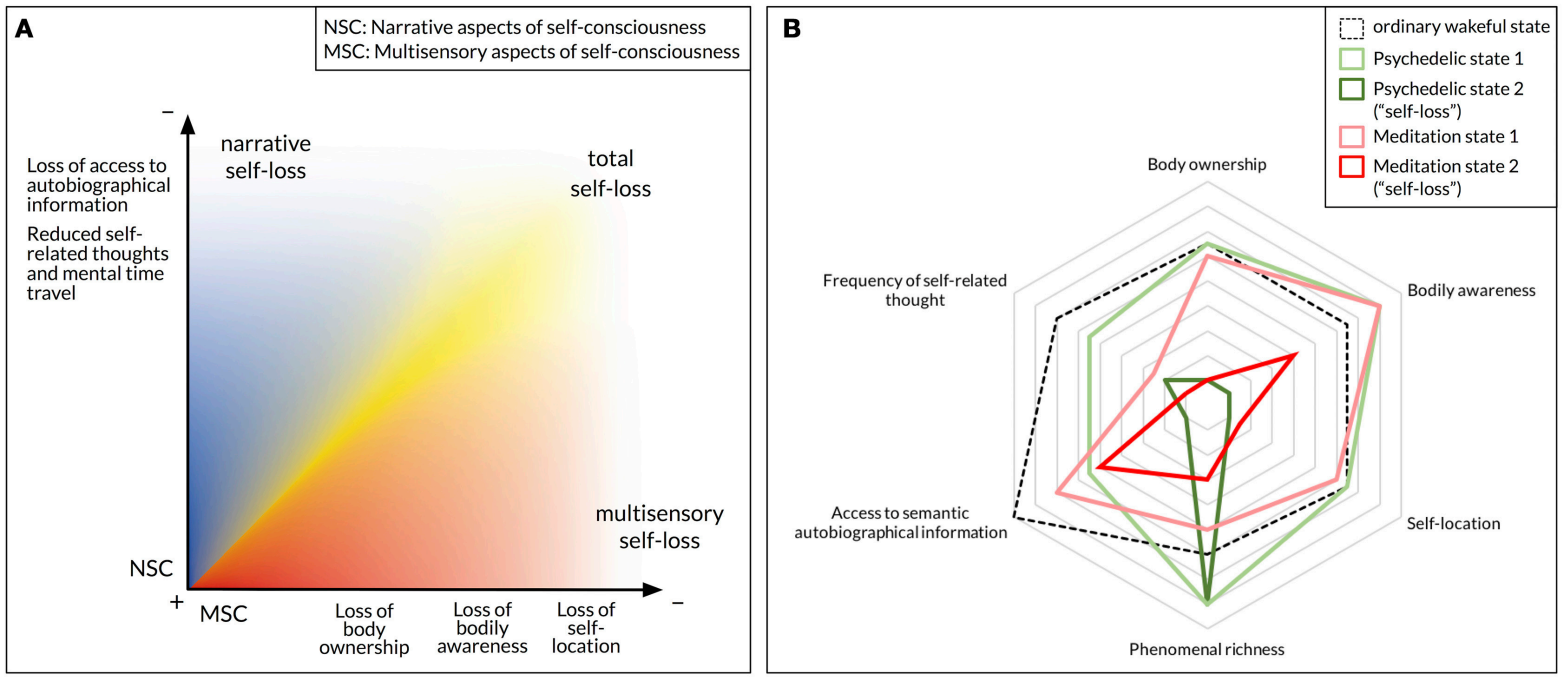
Multidimensional models of self-loss in global states of consciousness. **(A)** A simplified two-dimensional model of self-loss. The X-axis represents the degree to which multisensory aspects of self-consciousness are disrupted, and the Y-axis represents the degree to which narrative aspects of self-consciousness are disrupted. The color gradients represent the gradual disruption of narrative aspects (blue), multisensory aspects (red), or both (yellow) within the two-dimensional state space of altered states of consciousness induced by meditation and psychedelics could theoretically be plotted. This two-dimensional model can be conceived as a conceptual sketch that reduces the dimensionality of the notion of self-consciousness to two orthogonal principal dimensions, somewhat similarly to Principal Component Analysis. The shortcomings of this simplified model are tentatively addressed in the more complex **(B)**. **(B)** A tentative multidimensional model of self-loss. Global states of consciousness are plotted on the radar chart according to their score on six dimensions (using an arbitrary scale), representing the degree to which they involve (1) a sense of body ownership, (2) awareness of bodily sensations, (3) awareness of spatial self-location, (4) rich phenomenology, (5) access to semantic autobiographical information, and (6) self-related thoughts. Regions in the radar chart represent idealized examples of global states of consciousness, including an ordinary state during wakefulness (dotted black line), two examples of meditation-induced states and two examples of drug-induced states. The region in pink is an example of a typical meditative state with increased bodily awareness (via attentional focus on the breath), slightly decreased overall phenomenal richness (via visual-auditory deprivation) and decreased frequency of self-related thoughts. The region in red is an example of a “selfless” state described by experienced meditators, with a cessation of self-related thought, a loss of body ownership, agency and self-location, and significant reductions in bodily awareness and phenomenal richness. The region in light green is an example of a state induced by a moderate dose of psychedelic drugs such as LSD or psilocybin, with increased bodily awareness (modulated by salient and unusual bodily sensations) and increased phenomenal richness (via decreased sensory gating and vivid perceptual abnormalities). The region in dark green is an example of drug-induced ego dissolution with a loss of narrative and multisensory aspects of self-consciousness, but rich sensory content. **(B)** illustrates how even states of “total self-loss” as represented by a single region on **(A)** can differ from a phenomenological perspective between meditation (red line) and psychedelics (dark green line). In addition, it shows that some states of consciousness induced by both meditation practice and psychedelics can also score *higher* than baseline on certain dimensions of self-consciousness, in particular bodily awareness. Overall, states of “self-loss” are the exception rather than the norm for both modes of induction. Finally, it should be noted that the phenomenology of altered states induced by meditation and psychedelics may considerably change over time, sometimes very quickly; consequently, the idealized states plotted on this figure should be considered as phenomenological “snapshots” at a given time. For example, “Psychedelic state 1” (light green line) could be part of the same drug-induced experience as “Psychedelic state 2” (dark green line), assuming that the phenomenology dynamically shifts toward the peak of the experience (during the transition to drug-induced ego dissolution).

In what precedes, we have suggested that some meditation practices and some psychedelic substances can disrupt self-consciousness in different ways, or more precisely can disrupt different aspects or components of self-consciousness. This analysis stems from the assumption that self-consciousness is not a simple or unidimensional construct, as many other authors have emphasized (Blanke and Metzinger, 2009; Gallagher, 2013; Metzinger, 2014; Zahavi, 2014). We have proposed to organize different components of self-consciousness into two main categories, roughly equivalent to the influential dichotomy between “narrative” and “embodied” selfhood. Unlike some models of the narrative/embodied self-distinction, however, we have suggested that each of these dimensions can be modulated by meditation and drugs in different ways, and to different degrees. The resulting picture can be simplified in the form of a two-dimensional model of “self-loss” or “ego dissolution,” which is etiology-independent insofar as both meditation-induced and drug-induced altered states of consciousness could be located within this model, and overlap in some cases (Figure 1A).

This two-dimensional model is greatly simplified, because it suggests that the loss of body ownership, bodily awareness and self-location are all degrees of self-loss that can be ordered along the same dimension. Available evidence does suggest that this is often the case: for example, patients with somatoparaphrenia lack body ownership without lacking bodily awareness and self-location; individuals undergoing bodiless dreams, asomatic out-of-body experience and some psychedelic states (e.g., DMT-induced states) lack both body ownership and bodily awareness, but not necessarily self-location; and finally full-blown drug-induced ego dissolution, particularly after administration of 5-MeO-DMT, appears to involve the loss of all three components. Nonetheless, as we have mentioned, it is unclear whether meditation-induced states can involve a complete loss of bodily awareness (including interoceptive awareness), while it does seem that they can involve a loss of self-location. Thus, this two-dimensional model should be considered at most as a helpful idealization which reduces the dimensionality of the notion of self-consciousness. A more complex and accurate model would probably involve more dimensions (Figure 1B; see Bayne et al., 2016 on multidimensional accounts of global states of consciousness in general). In our example, we have represented global states of consciousness within a six-dimensional state space which models various cognitive and multisensory features of self-consciousness as independent dimensions (although systematic correlations are possible). Furthermore, this complex model takes into account another important variable to identify phenomenally distinct forms of self-loss, namely the overall richness or sparsity of phenomenology or the “bandwidth” of conscious contents. This parameter is relevant because many drug-induced states, including some of those reported as instances of drug-induced ego dissolution, have a rich sensory and emotional phenomenology, contrary to meditation-induced instances of self-loss. A more sophisticated model could perhaps take into account additional parameters, such as the sense of agency (see the end of section Spatial self-location above). Meditation-induced states involve intense attentional control at least during certain stages of the practice, unlike drug-induced states, although it is not obvious that this always results in a greater sense of agency in meditation than in psychedelic states. States of “objectless” or “pure consciousness” which lack ordinary phenomenal content, such as those induced by *Samadhi* practice and perhaps by certain psychedelics like 5-MeO-DMT, should be represented in the central region of this model, scoring the lowest in most if not all dimensions. Finally, it should be noted that “access to autobiographical information” is not on par with other dimensions of our tentative model, insofar as it is a functional and dispositional feature rather than phenomenological feature of conscious episodes. Nonetheless, as discussed in section Alterations of self-consciousness induced by meditation and psychedelics, the inability to retrieve semantic autobiographical information may occasionally be associated with a specific phenomenology of retrograde amnesia which differs from the mere cessation of self-related thoughts.

Although we have specifically focused on disruptions of self-consciousness in this paper, it is important to underline that both meditation and psychedelics can not only inhibit various aspects of self-experience, but may also *increase* their salience in other cases. In particular, meditation techniques focusing on the awareness of the breath, as well as psychedelic drugs such as LSD and psilocybin, may temporarily increase awareness of bodily sensations. This apparent paradox is resolved when one considers the many parameters that may modulate the phenomenology of meditation practice and psychedelic states. As we have insisted, there are many different styles of meditation, and states of “self-loss” are usually reported by highly experienced individuals trained in specific traditions. Moreover, the phenomenology of a single meditation session unfolds in distinct phases, with the occasional experience of self-loss being the culmination of this succession of phase transitions (Yamashiro, 2015; see Ataria et al., 2015; Dor-Ziderman et al., 2016). Similarly, the phenomenology of psychedelic states may be modulated by a number of variables, including: the particular drug used (e.g., the effects of 5-MeO-DMT appear to differ from those of other psychedelics), dosage (high doses being more likely to lead to ego dissolution), context of use (immobility and sensory deprivation may modulate the loss of body awareness and self-location) and finally pharmacodynamics (the effects of a drug evolve across time, with experiences of self-loss occurring at the peak, if at all). In Figure 1B, we have represented four examples of global states of consciousness induced by meditation and psychedelics to emphasize these nuances. Only two of these states (represented in red and dark green respectively) may be described as involving experiences of “total self-loss” as depicted on Figure 1A, although they do not completely overlap in the multidimensional state space. We have tentatively summarized in Table 4 below how such states of self-loss might differ between meditation and psychedelics.

**Table 4:** Summary of overlap and differences between meditation-induced and drug-induced states of “total self-loss”

	**Self-loss in psychedelic states**	**Self-loss in meditation**
Body ownership	**– – –**	**– – –**
Bodily awareness	**– – –**	**– –**
Self-location	**– – –**	**– – –**
Phenomenal richness	+ + + (except 5-MeO-DMT)	**– –**
Self-related thoughts	**– –**	**– – –**
Access to semantic autobiographical information	**– –**	**–**

The upshot of this discussion is that there is no such thing as “self-loss” or “ego dissolution” in absolute terms. Future research could develop new tools to assess in a more fine-grained way the features of conscious states described as involving a loss of one's sense of self. As an example, neuroimaging studies of psychedelics have often used a single questionnaire item (“I experienced a disintegration of myself or ego”) to measure drug-induced ego dissolution and correlate its intensity with neurophysiological observations (Muthukumaraswamy et al., 2013; Carhart-Harris et al., 2014, 2016b; Lebedev et al., 2016; Tagliazucchi et al., 2016). While this strategy has yielded very interesting results, it does not allow for the discrimination between several kinds of disruption of self-consciousness. The recent Ego Dissolution Inventory, which has been psychometrically validated, does not discriminate between the loss of narrative and embodied or multisensory aspects of self-consciousness either (Nour and Carhart-Harris, 2017). Future questionnaires focusing on alterations of self-consciousness could include both items related to disruptions of self-related thoughts and mental time travel, and items related to body ownership, bodily awareness and self-location. A psychometric analysis of subsequent item ratings could test whether these items can be grouped into two orthogonal factors, namely narrative and embodied self-loss.

It is also important to underline that evidence from subjective reports should be treated with caution. Although the reports discussed in this paper show a remarkable convergence and can be treated as preliminary evidence for the disruption of body ownership, bodily awareness and self-location in meditation and psychedelic states, alternative interpretations are available. First, one could refuse to take these self-reports at face value on the ground that they could be systematically unreliable and confabulatory. This is rather unlikely, however, given the convergence of reports from different groups of subjects in different conditions, as well as the consistency of reports with questionnaire data. Moreover, there is no reason to suspect that either meditators or volunteers participating in studies of psychedelic drugs are particularly prone to confabulation, and their reports are less problematic than those of patients suffering from delusions such as the Cotard syndrome (Billon, 2016). In addition, the microphenomenological interview used to collect some of these reports is designed to minimize the risk of confabulation and theoretical contamination (Petitmengin, 2006; Petitmengin and Lachaux, 2013). Finally, there is some evidence that meditative experience predicts introspective accuracy (Fox et al., 2012; Baird et al., 2014). Nonetheless, it should be noted that the evidence discussed in this article is still tentative insofar as most studies of the effects of meditation and psychedelics rely on a limited sample size.

A second alternative interpretation of the reports discussed in the previous sections would emphasize that the experience of loss is not equivalent to the loss of experience. In other words, the fact that subjects report having a sense of *losing* ownership over their body or awareness of their spatial location does not necessarily entail that body ownership or self-location were part of their overall phenomenology at baseline (i.e., prior to meditating or drug administration). While this idea is not incoherent, we have provided independent reasons to believe that at least bodily awareness and spatial self-location are part of the content of ordinary conscious experience; in other words, there is evidence that we usually experience bodily sensations, and have some awareness of our relative location with respect to our perceived environment. While we acknowledge that the existence of a specific phenomenology of body ownership is more controversial, we have also mentioned some clinical and experimental evidence from somatoparaphrenia, autoscopic phenomena, the rubber hand illusion and full-body illusions suggesting that some feature(s) of the ordinary experience of neurotypical individuals are associated with the experience of one's body as one's own.

In order to address the limitations of self-reports, future research could use implicit and behavioral measurements to circumvent the risk of introspective biases. For example, researchers could investigate the representation of trunk-centered peripersonal space, which has been shown to encode self-location (Blanke et al., 2015; Noel et al., 2015; Serino et al., 2015), both during meditation and after psychedelic intake. A plausible prediction is that the boundaries of the full-body (trunk-centered) peripersonal space are blurred both during some instances of drug-induced ego dissolution and some forms of meditation described as a loss of self-location and body boundaries (see Millière, 2017).

## Long-term outcomes of altered self-consciousness

### Selflessness as a trait

Accumulating evidence shows that meditation's state effects linger into daily life, to become long-term, trait alterations (Cahn and Polich, 2006). These trait effects include alterations in resting state function or connectivity, as well as structural changes, compared to control groups (Fox et al., 2014; reviewed in Tang et al., 2015). A full review of all the accumulated evidence is beyond the scope of this paper. Instead, here we focus on trait-effects of alterations in self-consciousness, either from the neurophysiological (underlying mechanisms, specifically regarding the DMN) or first-person perspective.

Several studies have provided evidence that meditation practitioners, compared to controls, exhibit reduced resting state DMN activity and connectivity, either using electrophysiology (Berkovich-Ohana et al., 2012, 2013) or fMRI (Hasenkamp and Barsalou, 2012; Berkovich-Ohana et al., 2016). Specifically, resting state functional connectivity analyses showed that following 8-weeks of mindfulness-training, participants demonstrated significantly increased functional connectivity of the anterior DMN region with the auditory/salience network (Kilpatrick et al., 2011). Similarly, long-term mindfulness practitioners showed compared to controls increased functional connectivity between DMN and visual regions (Berkovich-Ohana et al., 2016). In other studies, proficient mindfulness meditators showed increased functional connectivity between the DMN and fronto-parietal control network compared to novices (Brewer et al., 2011; Hasenkamp et al., 2012; Taylor et al., 2013), although there is some evidence of increased anti-correlation between the DMN and a task-positive network during focused attention meditation compared to the resting state (Josipovic et al., 2012), or the DMN and visual regions following MBSR (Kilpatrick et al., 2011). While fMRI studies largely show that mindfulness meditation practice is associated with reduced DMN activity, the related structural effects in the DMN are less clear (recently reviewed by Fox et al., 2014). Specifically, several neuroanatomical studies reported changes in PCC gray matter thickness: while a few studies indicate reduction in meditators relative to controls (Kang et al., 2013), another study failed to find group differences (Grant et al., 2013), and yet another reported gray matter increases following a short 8-weeks mindfulness meditation intervention (Hölzel et al., 2011). Initial evidence suggests that such changes in DMN functioning are indeed related to a reduced tendency to engage in self-referential processing. One earlier study (Farb et al., 2007) showed that after an 8-week mindfulness intervention, participants were able to disengage from a “narrative self-focus” in the trait judgment task (typically employed to investigate self-referential processes, cf. Northoff et al., 2006), as evidenced by more pronounced reductions in the DMN compared to the control group. Similarly, another study found reduced bias in neural responses to the self vs. an other's face for long-term meditators compared to controls (Trautwein et al., 2016).

Less is known about whether meditation practice affects multisensory aspects of self-consciousness in a trait-like manner. Studies investigating neural and phenomenological state effects of meditation suggest that long-term meditators are capable of flexibly modulating and reducing bodily self-awareness (Ataria et al., 2015; Dor-Ziderman et al., 2016). However, controlled studies have not yet systematically assessed whether this is an outcome of long-term practice (some effects also seem to occur in novice practitioners, cf. Dambrun, 2016) and to which degree such flexibility affects daily life functioning.

There is no evidence that the experience of drug-induced ego dissolution may have long-term effects on narrative and multisensory aspects of self-consciousness, such as a reduction of self-related thoughts. This question may be answered by future longitudinal studies.

### Relation to therapeutic outcomes, well-being and prosociality

While the long-term consequences of ego dissolution on self-consciousness are uncertain, there is preliminary evidence that drug-induced alterations of self-consciousness may mediate therapeutic outcomes. The current model of psychedelic-assisted therapy originated in the 50's and 60's (Pahnke et al., 1971; Dyck, 2006). In this model, subjects receive a high dose of a psychedelic drug in a supportive environment, with the aim of experiencing a mystical-type or “peak” experience (Maslow, 1959; Stace, 1960). This experience is conceived as a “unitive” state involving the loss of self-world boundaries, as described in sections Alterations of self-consciousness induced by meditation and psychedelics and Pure consciousness, non-dual awareness and total selflessness. The therapeutic model suggests that this experience mediates long-term outcomes (Pahnke et al., 1971; Grof et al., 2008; Majić et al., 2015; Richards, 2015). In recent years, many controlled studies have shown that that the magnitude of the mystical-type experience predicts positive psychological outcomes for depression, addiction, palliative care, and general well-being (O'Reilly and Funk, 1964; Klavetter and Mogar, 1967; Pahnke et al., 1971; Kurland and Grof, 1972; Richards et al., 1977; Griffiths et al., 2006, 2016; MacLean et al., 2011; Garcia-Romeu et al., 2014; Bogenschutz et al., 2015; Ross et al., 2016; Johnson et al., 2017; Roseman et al., 2018). Furthermore, a recent analysis of the psychedelic experience in uncontrolled environments suggests that mystical-type experiences predict changes in well-being (Carhart-Harris et al., 2018). It is also suggested that mystical-type experiences are linked to the positive emotional outcomes of meditation (Russ and Elliott, 2017).

Two variables appear to predict the occurrence of mystical-type experience in both meditation and psychedelics: trait absorption and surrender state. Absorption (Tellegen and Atkinson, 1974) is a trait characterized by the disposition to have episodes of “total” attention to one's representational resources. Subjects with higher absorption tend to have stronger mystical-type experiences both under psychedelics (Studerus et al., 2012) and in meditation (Russ and Elliott, 2017). The second variable is the pre-experience state characterized by the disposition to “let-go” or “surrender” to whatever experience comes, sometimes called “surrender state” (Richards, 2015). This state is not only related to the subject's personality but also to interactions with the environment (e.g., trust toward the therapist). Higher ratings of willingness to surrender are associated with stronger mystical-type experience in both psychedelic experiences (Carhart-Harris et al., 2018) and meditation (Russ and Elliott, 2017).

Do experiences of “self-loss” induced by meditation or psychedelics have a relevance for therapeutic outcomes or general well-being? There is preliminary evidence that the therapeutic effects of psychedelics for treatment-resistant depression is mediated by meaningful “breakthrough” experiences, which may include ego dissolution (Roseman et al., 2018). By contrast, long-term effects of contemplative practices on well-being do not appear to be necessarily mediated by intense experiences, but rather by training of different cognitive mechanisms, such as attentional control mediating meta-awareness of mind-wandering. Interestingly, one suggested mechanism is a (gradual) shift in the perspective on the self-described as “detachment from identification with a static sense of self” (Hölzel et al., 2011). In the Buddhist tradition, this process is thought to be a crucial factor for the attainment of stable well-being (Olendzki, 2006; Shiah, 2016). Some psychometric research supports this link (Dambrun, 2017). Moreover, a related construct called “decentering” has been found to mediate treatment effects of mindfulness based interventions (reviewed in Bernstein et al., 2015). Anecdotal reports and traditional sources do also claim that peak meditative experiences involving a loss of the sense of self can have lasting effects on well-being (Austin, 2009); however, perhaps due to the difficulty to induce such experiences in the lab, they have not been systematically investigated. In a first qualitative survey on experiences associated with long-term meditative practice, changes in the sense of self, including narrative and bodily levels were frequently reported (more than 75%) (Lindahl et al., 2017). Of note, the affective response to these experiences was not always positive, but ranged “from neutral curiosity, to bliss and joy, to fear and terror” (p. 20). Interestingly, ayahuasca administration was also found to produce after 24 h a significant reduction in judgmental processing of experiences measured by the Five Facets Mindfulness Questionnaire (FFMQ), as well as a significant increase in decentering ability measured by the Experiences Questionnaire (EQ) (Soler et al., 2016). Thus, it is possible that the medium- to long-term effects of meditation and psychedelic experiences involve a reduced tendency to be personally and emotionally engaged in one's thoughts and feelings, such that “the contents of consciousness are less filtered through considerations of self-relevance than is usual” (Letheby and Gerrans, 2017, p. 7).

A further question worth investigating in future research regards the specific kind of “self-loss,” if any, which may mediate long-term therapeutic outcome and increased well-being. For example, are such effects more likely to be mediated by the loss of narrative or multisensory aspects of self-consciousness? Given the potential link between mind-wandering and unhappiness (Killingsworth and Gilbert, 2010), it is intriguing to speculate that increased control of spontaneous thoughts might mediate increased well-being in experienced meditators, which would indicate that the disruption of narrative aspects of self-consciousness may have a positive effect, to some extent. At this stage, however, this speculative hypothesis remains unsubstantiated by significant evidence.

Besides individual well-being, one potential outcome of meditative practice is an increase in empathy and compassion, which are regarded as antecedents of prosocial behavior. There is indeed evidence that meditation fosters these aspects of prosociality (Condon et al., 2013; Ashar et al., 2016; see Kreplin et al., 2018 for a recent meta-analysis; Leiberg et al., 2011; Weng et al., 2013). Importantly, it has been hypothesized that these effects rely on a change in the sense of self. For example, Dambrun and Ricard (2011) suggested that meditation can support a shift from self-centered to selfless functioning, characterized by “a weak distinction between self and others, and self and the environment as a whole,” which in turn “is closely related to characteristics such as altruism, kindness, respect, empathy, compassion and the search for harmony” (Dambrun and Ricard, 2011, p. 140). Providing evidence for one part of this relationship, recent studies demonstrated that manipulation of bodily levels of self-awareness can affect empathy and social cognition (Maister et al., 2013, 2015). However, the link to meditation is still largely hypothetical, although one study found that reduced self-related processing in long-term meditators was correlated with increased trait levels of self-reported compassion (Trautwein et al., 2016). Intriguingly, the administration of a high dose of psilocybin 1 or 2 months after a program of meditation practice was found to occasion enduring trait-level increases in prosocial behavior (Griffiths et al., 2018). This finding raises the question of whether psychedelic intake may potentiate in certain cases the putative long-term effects of meditation on prosociality. In summary, it is possible that there is a link between “selflessness” as a conscious episode lacking self-consciousness and trait increase in prosocial behavior, but these two constructs should not be conflated. Future research could determine whether such an association exists for meditation training and perhaps psychedelic use, and get clearer on the mechanisms involved.

## General conclusion

There is converging evidence that high doses of psychedelic drugs and certain forms of meditation practice for highly experienced practitioners can produce strong, short-term, and reversible disruptions of self-consciousness. However, drug-induced and meditation-induced experiences of “self-loss” are not uniform, and can be decomposed in terms of alterations of various aspects or dimensions of self-consciousness. These include “narrative” aspects, such as the inhibition of self-related thoughts and self-related mental time travel, and the loss of access to autobiographical information, as well as bodily and multisensory aspects, such as the loss of body ownership, bodily awareness and self-location. Questionnaire data, subjective reports and neurophysiological results suggest that some of these aspects might be independently modulated in the global states of consciousness that can be induced by meditation and psychedelic drugs. Thus, the notion of self-consciousness can be construed as a multidimensional construct, and consequently “self-loss” or “ego dissolution” should not be conceived as a simple graded phenomenon ordered along a single dimension.

Moreover, even forms of putative “total” self-loss involving the radical disruption of both narrative and multisensory aspects of self-consciousness are best thought of as a family of states which can differ from a phenomenological perspective with respect to variables that are not directly related to self-consciousness. Indeed, strong forms of drug-induced ego dissolution may involve a very vivid and rich sensory phenomenology, perhaps as a result of decreased sensory gating, while available evidence on some “selfless” states induced by meditation suggests that their phenomenal content is very sparse (e.g., in states of so-called “pure consciousness” achieved in *Samadhi* practice).

One potential limitation of this analysis is that empirical data remains too sparse to reliably determine the phenomenological and neurophysiological specificity of the global states of consciousness under consideration. For example, it remains difficult to assess in what respect conscious states induced by *Samadhi* practice really differ from states induced by other meditation practices or psychedelic drugs. Few controlled studies have investigated the experience of self-loss in meditation or drug-induced states, and those which have done so have limited sample sizes. Another potential issue is the interpretation of self-reports. It is notoriously difficult to gather reliable evidence about subjective experience, and this is all the more problematic with altered states of consciousness often deemed ineffable.

Future research on this topic could focus on developing more fine-grained psychometric tools to validate and measure various dimensions of self-consciousness, and their respective disruptions in altered states of consciousness. Furthermore, researchers could seek to correlate reports gathered from questionnaires not only with neuroimaging data, but also with independent implicit measurements. As an example, the disruption of spatial self-location might be associated with a dissolution of the boundaries of the trunk-centered peripersonal space, which can be measured using an established psychophysical paradigm (Noel et al., 2015). Finally, future research could investigate the possible relationship between temporary disruptions of self-consciousness induced by meditation and psychedelics, and long-term changes in cognitive processing, personality traits and prosocial behavior. While it is important to avoid conflating states of self-loss with “selflessness” as a trait or moral construct, it is possible that correlations might exist between these distinct notions, although perhaps only for a subset of highly experienced meditators or drug-users. More research is needed to provide answers to these outstanding questions.

## Author contributions

All authors listed have made a direct contribution to the work. RM has written the first draft of the manuscript, with the exception of the final section Long-term outcomes of altered self-consciousness. RC-H, LR, F-MT and AB-O have written the latter, and have completed the section The neuroscience of meditation and psychedelics: an overview. All authors listed have contributed to subsequent revisions of the manuscript.

### Conflict of interest statement

The authors declare that the research was conducted in the absence of any commercial or financial relationships that could be construed as a potential conflict of interest. The reviewer MC and handling Editor declared their shared affiliation.

## Footnotes

^1^By “self-consciousness,” we refer in this article to a subject's consciousness of themselves – also called “sense of self,” “self-awareness,” “phenomenal selfhood,” or “self-experience.” Importantly, “self-consciousness” does not refer here to the social awareness of being the object of the observation of others.

^2^From a methodological perspective, disruptions of self-consciousness induced by meditation might be more challenging in terms of laboratory production, while disruptions of self-consciousness following the administration of psychedelic drugs might be more strictly dose-dependent and therefore easier to produce in a controlled setting. In a similar vein, the choice of baseline conditions and control groups is a multi-faceted issue in meditation research, often limiting interpretability, whereas placebo-controlled designs are the default for studies on psychedelics. Nonetheless, true placebo control is difficult to achieve with psychedelics, given their vivid and characteristic subjective effects.

^3^To be specific, meta-awareness of mind-wandering episodes may crucially involve *de se* thought, such as the thoughts “My mind is wandering” or “I need to refocus my attention on the breath.” Here, we are referring to what may happen with experienced meditators when this cycle of mind-wandering and meta-awareness comes to a halt, if only for a brief period, giving way to an episode of thought-free awareness.

^4^While it is possible that such drug-induced episodes are only partially integrated into autobiographical memory (similarly to dreams), these reports specifically suggest that the episodes themselves may involve a loss of access to autobiographical information.

^5^See section Spatial self-location below for anecdotal reports and questionnaire data regarding states induced by 5-MeO-DMT.

^6^On the notion of egocentric frame of reference, see Klatzky (1998).

^7^In a predictive coding framework, in particular, it is reasonable to assume that the violation of expectations regarding one's bodily movements, rather than the match between those expectations and sensory feedback, modulates phenomenology. Accordingly, prediction error signals might give rise to a feeling of alienation from one's action, but the absence of prediction error signals should not be associated with a positive feeling of agency (Fletcher and Frith, 2009; although see Hohwy, 2016 for a predictive coding account of the sense of agency). This explanatory model can be extended to thoughts to account for schizophrenic thought insertion without appealing to a loss of the sense of agency (Parrott, 2017). However, it should be acknowledged that the existence of a positive phenomenology of agency might be more in line with the active inference framework, according to which organisms minimize prediction error not only by updating prior expectations, but also through action (Friston, 2013). Within this framework, the sense of agency may result from the process of modeling the effects of the selective sampling of sensations through action (Limanowski and Blankenburg, 2013).

^8^Importantly, the notion of subject of experience at play in this claim is very thin—it does not carry more substantial commitments about subjects being Cartesian substances enduring in time, or identical to organisms or parts of organisms. Rather, the claim merely stems from conceptual analysis of the notion of experience. Accordingly, a thin subject of experience is simply the bare locus of an experience; an experience cannot occur without there being something it is like for such a thin subject to have it, and conversely a thin subject cannot exist without the occurrence of an experience whose subject it is (see Peacocke, 2014; Strawson, 2017). One might wonder whether this supposedly uncontroversial claim is compatible with the Buddhist “no self” doctrine. This is a difficult question, not least because there are several interpretations of the “no self” doctrine. However, common interpretations of the “no self” view articulate two claims: a metaphysical claim, according to which there is no such thing as *the self* construed as a substance or enduring entity; and a phenomenological claim, according to which the sense of self is not a necessary and invariant feature of experience (see MacKenzie, 2016). Both points are compatible with the minimal claim that all experiences have subjects of experiences, in the thin sense outlined above.

^9^Note that this further assumption does not sit well with the proposals of Nida-Rümelin and Zahavi, according to whom “pre-reflective self-awareness” or “self-givenness” is a necessary component of all conscious experiences in general.

^10^One possibility, in line with the distinctions outlined in section Alterations of self-consciousness induced by meditation and psychedelics, is that NDA states may involve a minimal awareness of bodily sensations (e.g., interoceptive signals) without a sense of body ownership (an awareness of these sensations *as one's own*).

^11^To be clear, we are not denying that actual descriptions of NDA states from contemplative scholars may seem more consistent with the second interpretation, if they are taken at face value. Rather, we suggest that such an interpretation rests on an account of the phenomenology of ordinary experience which is at best ambiguous and at worst implausible—in so far as it seems to postulate the ubiquity of a *sui generis* “sense of mineness.” Accordingly, we put forward an interpretation of NDA states which appears to be more consistent with an evidence-based account of the multiple dimensions of the sense of self, as described in section Alterations of self-consciousness induced by meditation and psychedelics. It is also worth emphasizing that our purpose in this article is *not* to engage in the exegesis of historical sources from the Buddhist and Hindu traditions, but to offer a critical discussion of available evidence regarding global states of consciousness described as involving a dramatic loss of the sense of self.

^12^As previously noted, it is by definition impossible for a phenomenally conscious mental state to lack any phenomenal content. More realistically, states induced by *Samadhi* practice might lack *ordinary* phenomenal content.

^13^However, recent data suggests that TM practice does not involve focused attention either and relies on distinct mechanisms; given that TM is often described in terms of pure consciousness, this casts doubt on Josipovic's assumption that “objectless” meditation is a form of focused attention (Travis and Parim, 2017).

^14^Some narrative reports about 5-MeO-DMT from Erowid.org explicitly refer to *Samadhi* practice: “Everything I've read about enlightenment and the attainment of Samadhi makes perfect sense to me now. It has all become so obvious. During this part of the experience I am not me, I am simply awareness” (#88450), “an immediate complete dissolution of any identity and a merging into the oneness, timeless, pure awareness… similar in some ways with a previous *Samadhi* meditation experience” (#5804).
